# Consumer Acceptability of Animal-Source Food Alternatives: A Literature Review to Support the Transition to Sustainable, Healthy Diets

**DOI:** 10.1016/j.cdnut.2026.109513

**Published:** 2026-08-11

**Authors:** Stella Nordhagen, Ty Beal, Mario Herrero, Lynnette M Neufeld, Kristina Sokourenko, Maria Xipsiti

**Affiliations:** 1 Global Alliance for Improved Nutrition, Geneva, Switzerland; 2 Global Alliance for Improved Nutrition, Washington, DC, United States; 3 Cornell Atkinson Center for Sustainability, Cornell University, Ithaca, NY, United States; 4 Food and Nutrition Division, United Nations Food and Agriculture Organization, Viale delle Terme di Caracalla, Rome, Italy; 5 Ashley School of Global Development and the Environment, Cornell University, Ithaca, NY, United States

**Keywords:** alternative proteins, meat analogs, dairy analogs, protein transition, food choice

## Abstract

Animal-source foods (ASFs) have long been central to human diets but have come under increased scrutiny in recent years because of relatively large environmental impacts, animal welfare considerations, and some negative health impacts. One option for addressing these issues is (at least partially) replacing ASFs in the diet with alternatives to ASFs (Alt-ASFs): conventional or novel foods with the potential to be consumed as a substitute for conventional ASFs such as meat, eggs, dairy, and fish. However, the adoption of such products at scale depends on consumers purchasing, preparing, and eating them as part of their broader eating practices. This paper undertakes a structured narrative review informed by scoping review methods of consumer acceptability of alternative animal-free sources of serum (Alt-ASFs), separately considering Alt-ASFs made from plants, cells, insects, algae, and fungi. Two databases (Scopus, Google Scholar) were searched, covering articles published from 1 January, 2000 until 24 February, 2024 (the search date). A total of 5998 records were identified, and 61 were included in the full-text synthesis, covering 29 countries. The results show that acceptability varies significantly by Alt-ASF type. Key barriers across all categories include poor sensory qualities (e.g., taste, texture), limited access, high prices, unfamiliarity, and perceived inconvenience. Facilitating factors include perceived health and environmental benefits, though these alone rarely overcome the aforementioned barriers. Plant-based alternatives have the highest acceptance, whereas cell-based and insect-based alternatives have the lowest acceptance, although this varies across cultures and evidence is limited for some contexts. Although plant-based alternatives are often largely seen as merely less desirable due to sensory and convenience factors, cell- and insect-based alternatives may not even be seen as food by many consumers, who may express fear or disgust at eating them. Although food acceptability evolves over time through cultural, commercial, and policy influences, scaling Alt-ASF consumption will require both product improvements and consumer outreach.

## Introduction

Animal-source foods (ASFs) have long been and continue to be a central part of human diets. They contribute essential micronutrients and protein in highly bioavailable forms, can be part of healthy diets, and are deeply integrated into culinary traditions around the world [1,2]. Their nutrient density makes them particularly useful at certain stages of the life course, such as during pregnancy and early childhood [1]. However, they have come under increased scrutiny in recent years because of their relatively large environmental impacts [1,[3], [4], [5], [6]]. Terrestrial livestock are responsible for ∼40% of greenhouse gas emissions from food systems, or ∼12%–14% of total emissions [7,8], with aquaculture and fishing responsible for an additional 3% of total emissions [9]. As the effects of climate change continue to accelerate and increasingly affect quality of life around the world, reducing emissions is critical [10]. ASF production also uses a significant amount of land and water and releases contaminants that can harm local ecosystems [11]. These burdens are widely thought to be unsustainable, particularly for the highest-consuming populations and particularly given that global per-capita demand for ASF has grown by 35% in the past 25 y [12].

This, as well as concerns around negative health effects associated with high consumption of certain ASFs, excessive antibiotic use in livestock systems, and animal welfare issues, has led some to advocate for limiting consumption of ASFs as part of a transition to a more sustainable eating pattern, at least among the highest-consuming populations [2,3,13]. Reducing ASF consumption would likely entail at least partially replacing ASFs in the diet with alternatives to ASFs (Alt-ASFs), commonly referred to as “alternative proteins” or “alt proteins” and defined as conventional or novel foods with the potential to be consumed as a substitute for conventional ASFs such as meat, eggs, dairy, and fish. Although theoretically other foods not typically positioned as substitutes (e.g., vegetables, grains) could be consumed instead, at least some Alt-ASFs are likely needed to meet nutritional needs and align with consumer habits of having ASFs as part of their dietary behaviors.

The more rapidly a shift from ASFs to Alt-ASFs were to take place, at the largest scale, the greater the potential dividends in reduced environmental impact would be [14]. However, the adoption of Alt-ASFs at scale depends on consumers purchasing, preparing, and eating them as part of their broader eating patterns and practices. Consumer dietary behaviors are driven by many factors, such as food availability and affordability, but acceptability is a central one [15]. Food acceptability is defined for the purposes of this paper, adapting House et al. [16], as “the qualification of particular foods as appropriate, or even desirable, to eat, either for a particular individual or for a particular population”; it encompasses both the basic binary dimension of “would you eat this?” as well as the more complex “how *much* would you *like* to eat this?” (sometimes referred to as “desirability”). Acceptability is influenced by both individual characteristics (e.g., personal taste, allergies, medical conditions, and prior experiences) and sociocultural ones (e.g., typical meal patterns, traditional foods, what a culture defines as “food” or “not food”) and can be dynamic over time [16]. Where consumers have options, they generally do not eat foods they see as unacceptable. If Alt-ASFs are not acceptable to a large share of consumers, it is thus unlikely that they will be adopted widely enough to displace sufficient ASFs to significantly reduce ASFs’ abovementioned negative environmental impacts. Moreover, “cultural acceptability” is an integral component of the definition of a sustainable diet: thus, if certain Alt-ASFs are not culturally acceptable, they should not be considered a viable option for encouraging large-scale adoption of sustainable diets [17].

Understanding consumer acceptability of Alt-ASFs is thus crucial to inform actions to support a transition to sustainable and healthy diets (Although the acceptability of alternative ASFs to other food system actors [e.g., farmers, entrepreneurs, policymakers] is also relevant, here we focus only on consumer acceptability [henceforth simply “acceptability”].). This paper reviews the topic of consumer acceptability of alternative animal-free (Alt-ASFs) to inform future policy, programming, and research on Alt-ASFs and thus support an equitable food system transformation that is supportive of both human and planetary health. Specifically, the insights from this review can help determine which (if any) policy or programmatic efforts should be made to scale-up Alt-ASF production and consumption and for which specific types of Alt-ASFs. It can also inform what consumer education efforts should focus on, and it can help identify where and among whom shifts are most and least feasible, enabling more efficient use of resources. For product developers and food technologists, it can provide information on how products need to change to facilitate adoption.

## Methods

The review sought to answer the following research questions:

• What is the state of research to date on the topic of acceptability of Alt-ASFs?
• How does acceptability of Alt-ASFs compare across types of Alt-ASFs and to that of ASFs?

Across both questions, we sought to present a comprehensive examination across many different types of Alt-ASFs and study contexts. The first research question thus focuses particularly on understanding the extent to which the acceptability of different Alt-ASF types has been studied and where. This is important to structure future research efforts to be equitable and resource-efficient and also to inform the interpretation of the answer to the second research question: if most evidence were focused on a limited set of foods or contexts, it would be important to not overgeneralize from this when making recommendations.

Regarding the second research question, understanding which Alt-ASFs are the most (and least) acceptable to consumers is important, as different Alt-ASFs differ in their nutritional, environmental, and other properties [[18], [19], [20], [21]]. Decisions by policymakers, project implementers, and product designers should take these differences into account when prioritizing options—for example, focusing on those with the lowest environmental impacts—but must also understand the consumer perspective to make a balanced decision. Differences in acceptability of Alt-ASFs also affect the equity implications of a potential scale up: if Alt-ASF acceptability varies across countries or demographic groups, adoption and consumption rates can also be expected to vary—with potential implications for nutrition and health as well as the distribution of economic benefits and risks to Alt-ASF production. Considering a comprehensive set of potential Alt-ASFs (as opposed to, e.g., only plant-based options) and comparing among them is thus important. Considering how their acceptability compares with that of ASFs is also essential: Alt-ASFs can be eaten in place of ASFs or as additional elements in the diet, without an effect on ASF consumption, but for Alt-ASFs to play a role in shifting consumption patterns to be more environmentally sustainable, they would need to be replacing or displacing higher-environmental-impact foods, particularly ASFs.

We used the following definition of Alt-ASF: “a conventional or novel food or culinary ingredient with the potential to be consumed as a substitute for conventional ASFs like meat, eggs, dairy, fish, and seafood.” Although other terms, such as “alternative protein,” are commonly used [22], we use “Alt-ASF” because it is broad enough to cover a wide range of products and avoids the pitfalls of other terms—for example, it reflects that ASFs contain many dietary components and nutrients beyond protein [1] and that Alt-ASFs can include foods that are not direct analogs of ASFs (e.g., legumes as well as “veggie burgers”). We divide the Alt-ASF category into 6 subcategories, based on the main ingredient, as described in Table 1.

**TABLE 1:** Alt-ASF categories used to guide the review

Type	Definition	Typically positioned as an alternative to…
Plant-based Alt-ASFs	Alt-ASFs made from plants such as legumes, nuts, and seeds, including unprocessed or minimally processed foods such as beans, traditional processed foods such as tofu, and highly processed foods such as plant-based burgers or sausages	Dairy, egg, terrestrial animal meat, and aquatic animal meat
Algae-based Alt-ASFs	Alt-ASFs made from macroalgae sources like seaweed as well as microalgae sources like spirulina and chlorella	Aquatic animal meat
Fungi-based Alt-ASFs	Alt-ASFs made from fungal sources, specifically mycelium (i.e., the root-like structure of fungi) or mycoprotein (protein extracted from mycelium)	Terrestrial and aquatic animal meat
Cell-based Alt-ASFs	Alt-ASFs made in controlled environments either from animal cell cultures grown into a tissue (i.e., cell-based or cultivated meat), or via precision fermentation through which organisms such as yeast or bacteria produce a desired product (e.g., casein, whey), often aiming to be identical to an animal-source component	Terrestrial and aquatic animal meat (cell cultivation); egg and dairy (precision fermentation)
Insect-based Alt-ASFs	Traditional and novel foods made from edible insects, either whole or processed into various forms	Terrestrial animal meat
Composite Alt-ASFs	Alt-ASFs made from a combination of ASF and Alt-ASF, including any of the categories noted above	Terrestrial and aquatic animal meat

Abbreviation: Alt-ASF, alternative to animal-source food.

The definition and typology have been used to guide 4 other recent reviews on nutritional, environmental sustainability, price, and livelihood aspects of Alt-ASFs [[18], [19], [20], [21]]; using it here can help facilitate synthesis on acceptability alongside these other key topics related to Alt-ASF adoption.

This review was a structured narrative review informed by scoping review methods. Given our focus on a wide set of different Alt-ASF types, the large amount of literature published on the subject precluded a full scoping review, which became clear in the initial searches. However, we drew on practices for scoping reviews to strengthen the narrative review method, where feasible. This included using the methodological framework proposed for scoping reviews by Arksey and O’Malley [23] and further developed by Levac et al. [24] to guide the review, developing a protocol following the PRISMA—Extension for Scoping Reviews (PRISMA-ScR [25]), registering it with Open Science Framework (OSF) in February 2024, at the start of the search process (OSF.IO/U9SKR), and following PRISMA-ScR guidelines in reporting as far as possible.

This review included studies using qualitative, quantitative, and mixed-methods published in peer-reviewed journals, as well as review articles. The topic was expected to span diverse fields and disciplines, so the search strategy focused on nondiscipline-specific databases. Searches were carried out in Google Scholar and Scopus. Scopus was chosen as the primary search database because it has among the largest coverage of any nondiscipline-specific search engine, with extensive coverage of diverse fields and inclusion of a considerable amount of interdisciplinary and international research [26]. Google Scholar was chosen as a complementary search database because of its diverse coverage of disciplines and types of resources.

Search results were limited to English and to articles published from 2000 onwards. Each search included a set of terms covering various words for “Alt-ASF” and “acceptability.” For each search, at least one word from each of the 2 categories had to be included. Search functions supported vary between Scopus and Google Scholar, so the search approach varied slightly between the 2 databases, as described in detail in the Supplemental Materials, including full search terms. Searches were undertaken between 17 February, 2024 and 11 March, 2024. Because of the depth of the scientific literature, no efforts were made to include gray literature.

All identified studies were reviewed against preidentified inclusion and exclusion criteria through separate title, abstract, and full-text screening. Inclusion and exclusion criteria are summarized in Table 2. Of note, both studies that examined the acceptability of Alt-ASFs specifically as replacements for ASFs and those that examined the acceptability of Alt-ASFs on their own (i.e., in place of ASFs, in place of other foods, as an addition to the diet, or unspecified) were included.

**TABLE 2:** Study inclusion and exclusion criteria

To be included, studies needed to meet all the inclusion criteria	And none of the exclusion criteria:
• Discuss acceptability of Alt-ASFs, including composite proteins that mix ASFs and Alt-ASFs • Published paper in scientific journals indexed in the searched databases (at the full-text stage; for title and abstract screening, indexed book chapters and conference papers were also included) • Published in English • Published in or after 2000	• No full text available (there was no follow-up with authors to obtain full-text versions) • Product development and testing studies • Hypothetical tests of marketing messaging and similar • Studies on Alt-ASFs for uses other than human food (i.e., as animal feed or as inputs into biofuels, cosmetics, or chemicals) • Opinion and perspective pieces, without novel data or a review/synthesis

Abbreviation: Alt-ASF, alternative to animal-source food.

All articles passing abstract screening were included in the quantitative literature summary. However, because of the large number passing abstract screening (*n* = 410), a prioritization approach was used to identify 61 articles to include in the detailed synthesis. First, we included all systematic and scoping reviews. Second, for Alt-ASF that had been covered by a recent, strong structured review (i.e., cell-, fungus-, and insect-based foods), we included ≤10 articles per Alt-ASF type that: were published after the most recent search covered in the reviews, included primary data, had acceptability of Alt-ASF as their main focus, covered a general population, and did not have any obvious quality issues. Third, for the other Alt-ASF categories (plant- and algae-based), we included ≤10 additional articles per Alt-ASF, prioritizing first unstructured (narrative) reviews and then original studies that met the criteria elaborated in the prior sentence. Finally, we included all 3 additional studies on composite Alt-ASFs that met the abovementioned criteria.

**FIGURE 1:**
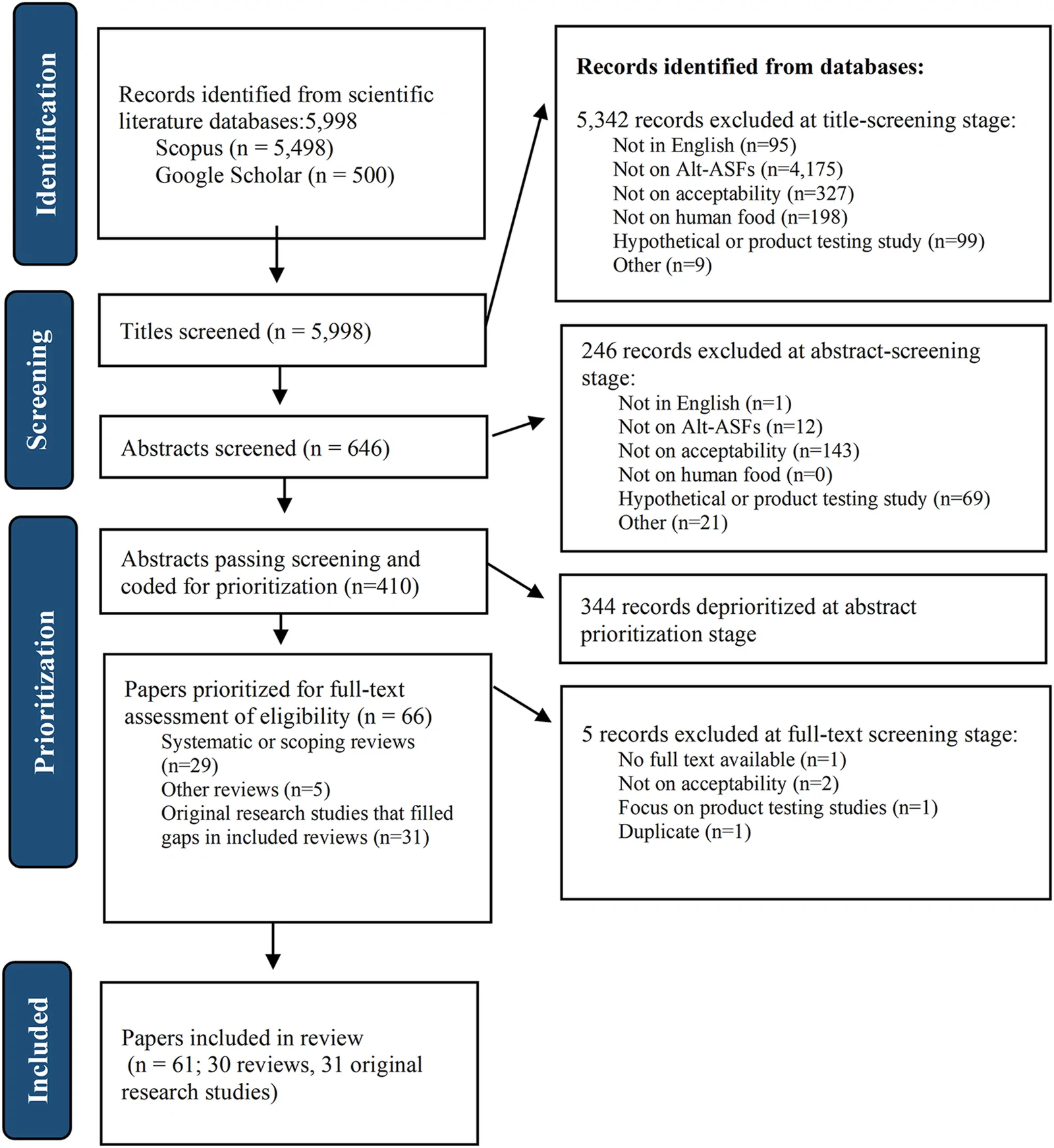
PRISMA flow diagram for the review. Adapted from reference [27] with permission. Alt-ASF, alternative to animal-source food.

This dual approach was used because of the large scale of the literature (making it infeasible to review all full-text studies in depth) and was designed to provide comprehensive coverage of all main types of Alt-ASFs while privileging higher-quality research types (e.g., systematic reviews), where available. However, it entails that this paper’s synthesis does not reflect all eligible studies identified during screening, and it does introduce bias, as there was substantial author discretion in choosing which studies to include. To mitigate this through transparency, full details on how the 61 studies were selected are provided in the Supplemental Materials, and a list of all 410 identified studies is also included in the Supplemental Materials. Extraction and synthesis followed a structured process, as detailed in the Supplemental Materials. In the synthesis, we grouped products into the 6 ingredient-based subcategories listed above. Figure 1 indicates the records identified and removed at each stage, with removal reasons, using a PRISMA flow diagram [27].

## Results: State of the Literature

**FIGURE 2:**
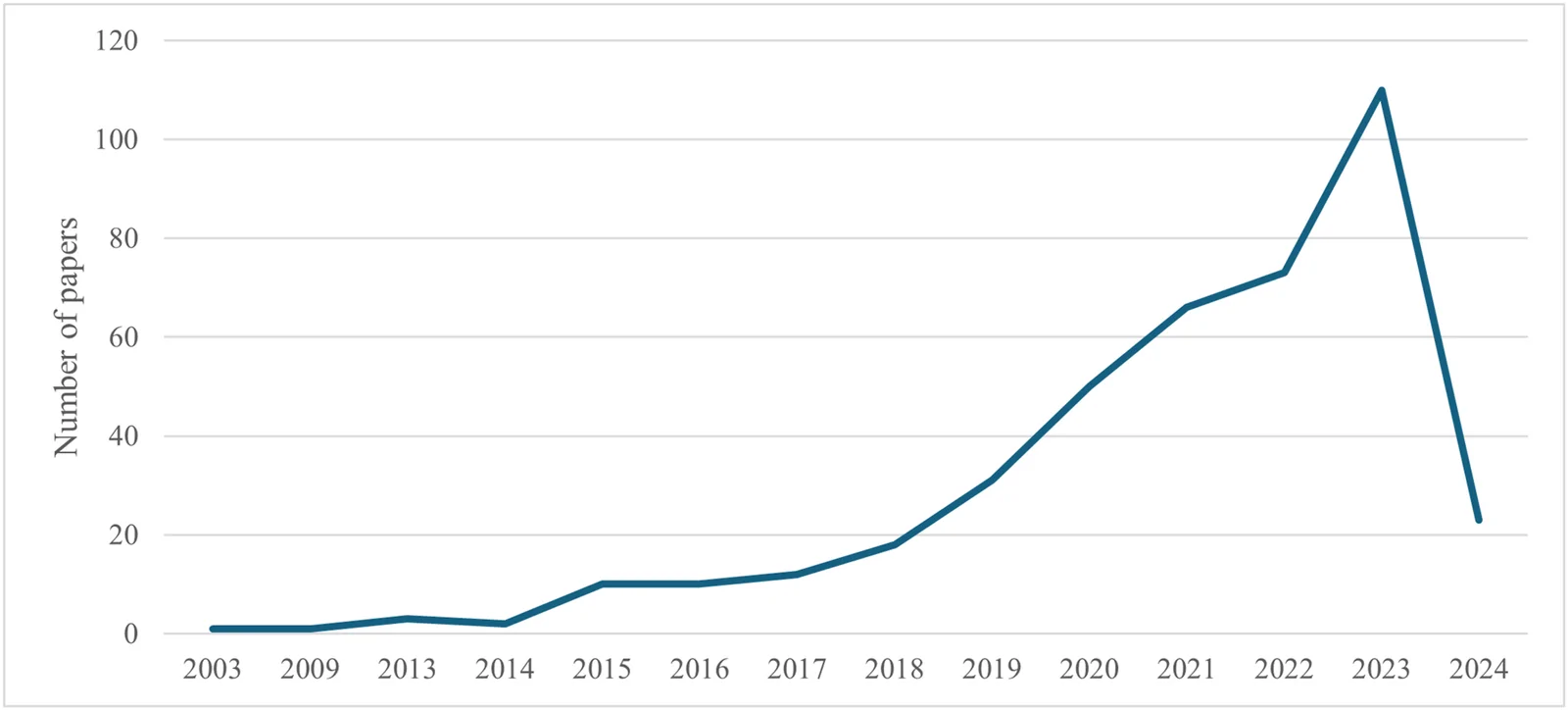
Distribution of studies over time (*n* = 410).

**FIGURE 3:**
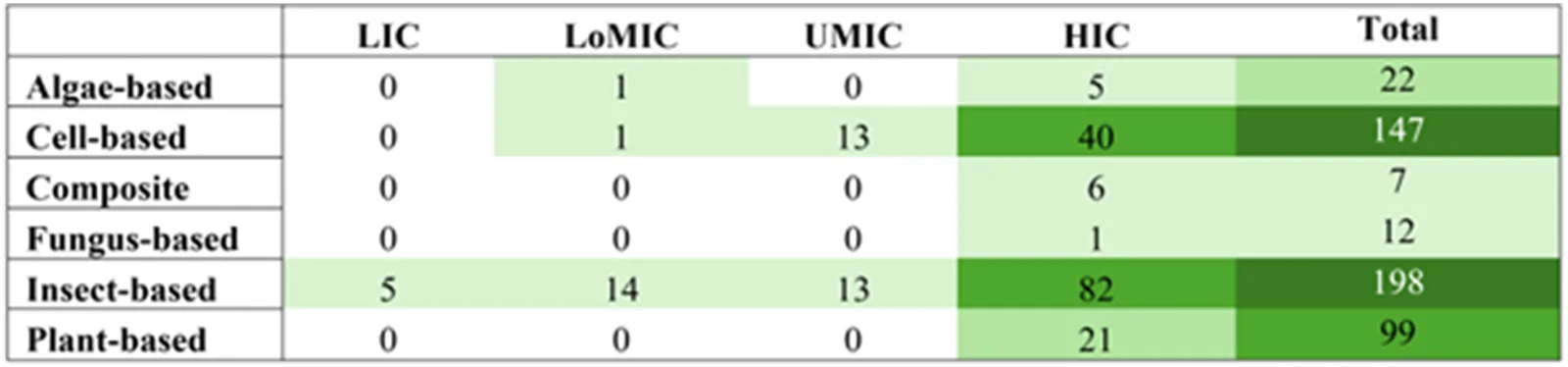
Number of studies identified, by Alt-ASF type and country income group. Note: The darkness of the green shading indicates the relative number of studies, with darker colors indicating more studies. The “total” column also includes studies with no geographic focus, and some studies covered multiple Alt-ASF types. Alt-ASF, alternative to animal-source food; HIC, high-income country; LIC, low-income country; LoMIC, lower-middle-income country; UMIC, upper-middle-income country.

From a total of 5998 titles identified through the searches, 646 were included in the abstract screening. A total of 410 articles passed the abstract screening and were coded for analysis. Of those 410 studies, all but 2 were published in 2013 or later, with 79% published in 2020 or later, and the largest share (*n* = 110, 26.7%) published in the most recent full year covered in the search (2023), indicating the novelty and growing interest in the topic (Figure 2). Considering geographic representation, one-third of studies were global, with no specific geographic focus, whereas 9 focused on “Western” countries and 1 on low- and middle-income countries (LMICs). For those with a geographic focus (*n* = 264), all regions were covered, but there was a strong bias toward Europe, followed by Asia and North America. Only one study (in Israel) focused on the Middle East and North Africa; all studies in Oceania were on Australia or New Zealand. As Figure 2 shows, studies with a specific geographic focus were biased toward high-income countries (HICs). Studies covered all Alt-ASF categories, but with a bias toward insect-based and cell-based Alt-ASFs. About 17% of studies covered multiple different Alt-ASF categories. Fungus-based foods were the least well-covered category. We defined the “cell-based” category as including precision-fermented dairy and egg as well as cell-based meat, but most studies focused on cell-based meat. Most plant-based food studies focused on plant-based meat analogs, but there were also studies on plant-based beverages, cheese, seafood, eggs, and minimally processed legumes. Studies in low-income countries and lower-middle-income countries mostly focused on insect-based foods, whereas those in upper-middle-income countries covered both cell- and insect-based foods, and those in HICs covered all types of Alt-ASFs (Figure 3).

Although the above analysis reflects patterns across all 410 coded studies, the next sections draw on the 61 articles included in full-text extraction (listed in Table 3 [[28], [29], [30], [31], [32], [33], [34], [35], [36], [37], [38], [39], [40], [41], [42], [43], [44], [45], [46], [47], [48], [49], [50], [51], [52], [53], [54], [55], [56], [57], [58], [59], [60], [61], [62], [63], [64], [65], [66], [67], [68], [69], [70], [71], [72], [73], [74], [75], [76], [77], [78], [79], [80], [81], [82], [83], [84], [85], [86], [87], [88]]) to analyze key themes of the literature, focusing on those emergent from reviews or multiple single studies. Of these 61 studies, 25 were global, 2 each focused on Europe and “Western” countries, 1 on Sub-Saharan Africa, and 33 on an individual country or set of countries, covering a total of 29 countries.

**TABLE 3:** Summary of all studies included in the review

First author surname	Year of publication	Title	Journal name	Country/countries of study	Alt-ASF focus	Alt-ASF focus (categorical)	Method summary
Alhujaili [28]	2023	Insects as food: consumers’ acceptance and marketing	Foods	Global	Edible insects	Insect-based	Systematic review
Appiani [29]	2023	Sensory properties and consumer acceptance of plant-based meat, dairy, fish and eggs analogs: a systematic review	Frontiers in Sustainable Food Systems	Global	Plant-based alternatives to meat, dairy, fish, eggs	Plant-based	Systematic review
Banovic [30]	2022	Enabling sustainable plant-forward transition: European consumer attitudes and intention to buy hybrid products	Food Quality and Preference	Denmark, Spain, UK	Composite: 50% beef, 50% plant-based ingredients (bean, pea, oat, rapeseed, and soy)	Composite	Quantitative
Bryant [31]	2018	Consumer acceptance of cultured meat: a systematic review	Meat Science	Global	Cell-based meat	Cell-based	Systematic review
Bryant [32]	2020	Consumer acceptance of cultured meat: an updated review (2018–2020)	Applied Sciences	Global	Cell-based meat	Cell-based	Systematic review
Chia [33]	2024	Complexity of consumer acceptance to alternative protein foods in a multiethnic Asian population: a comparison of plant-based meat alternatives, cultured meat, and insect-based products	Food Quality and Preference	Singapore	Plant-based meat alternatives, cultured meat, insect-based products	Plant-based; cell-based; insect-based	Quantitative
Cunha [34]	2023	Understanding the perception of edible insects	British Food Journal	Brazil	Eating insects as a general concept	Insect-based	Quantitative
De Cianni [35]	2023	A systematic review on drivers influencing consumption of edible mushrooms and innovative mushroom-containing products	Appetite	Global	Innovative mushroom-containing products	Fungus-based	Systematic review
Dean [36]	2022	Understanding key factors influencing consumers’ willingness to try, buy, and pay a price premium for mycoproteins	Nutrients	China, USA, France, UK, New Zealand, Netherlands, Brazil, Spain, Dominican Republic, Mexico, Indonesia, Pakistan	Mycoprotein	Fungus-based	Quantitative
Dean [37]	2023	Should I really pay a premium for this? consumer perspectives on cultured muscle, plant-based and fungal-based protein as meat alternatives	Journal of International Food and Agribusiness Marketing	China, USA, France, UK, New Zealand, Netherlands, Brazil, Spain, Dominican Republic, Mexico, Indonesia, Pakistan	Plant-based proteins, fungal-based proteins and cultured muscle tissue	Plant-based; cell-based; fungus-based	Quantitative
Eckl [38]	2021	Replacement of meat with non-meat protein sources: a review of the drivers and inhibitors in developed countries	Nutrients	Global	Non-meat protein sources	All	Scoping review
Embling [39]	2022	“Edible seaweeds” as an alternative to animal-based proteins in the UK: Identifying product beliefs and consumer traits as drivers of consumer acceptability for macroalgae	Food Quality and Preference	UK	Seaweeds and seaweed-containing products (specifically considered an energy bar, burger, pasta, sushi, juice drink, and a sweet type of kelp)	Algae-based	Quantitative
Fiorentini [40]	2020	Role of sensory evaluation in consumer acceptance of plant-based meat analogs and meat extenders: a scoping review	Foods	Global	Plant-based meat analogs and extenders	Plant-based; composite	Scoping review
Florença [41]	2022	The motivations for consumption of edible insects: a systematic review	Foods	Global	Edible insects	Insect-based	Systematic review
Gbejewoh [42]	2022	Planetary health and the promises of plant-based meat from a sub-Saharan African perspective: a review	Scientific African	Framed as being about Africa (SSA), but most included studies were conducted in the US, UK, Europe, and Canada	Plant-based meat analogs	Plant-based	Scoping review
Hartmann [43]	2017	Consumer perception and behavior regarding sustainable protein consumption: a systematic review	Trends in Food Science & Technology	Global	Cell-based; insect-based; plant-based products	All	Scoping Review
Henn [44]	2022	Willingness to replace animal-based products with pulses among consumers in different European countries	Food Research International	Denmark, Germany, Poland, Spain, UK	Pulses	Plant-based	Quantitative
Islam [45]	2023	The perception of consumers toward microalgae as an alternative food resource in bangladesh: a contingent valuation approach	Evergreen	Bangladesh	Spirulina tablet, powder, and supplemented foods	Algae-based	Quantitative
Jallinoja [46]	2016	Future of sustainable eating? Examining the potential for expanding bean eating in a meat-eating culture	Futures	Finland	Dried/canned beans	Plant-based	Quantitative
Klockner [47]	2022	Milk, meat, and fish from the petri dish—which attributes would make cultured proteins (un)attractive and for whom? Results from a Nordic survey	Frontiers in Sustainable Food Systems	Norway, Denmark, Finland	Cultured meat, fish, or dairy	Cell-based	Quantitative
Kouarfaté [48]	2023	A systematic review of determinants of cultured meat adoption: impacts and guiding insights	British Food Journal	Global	Cell-based meat	Cell-based	Systematic review
Kröger [49]	2022	Acceptance of insect-based food products in Western societies: a systematic review	Frontiers in Nutrition	Western countries	Edible insects and processed insect-based products	Insect-based	Systematic review
Kumari [50]	2024	Sensory evaluation of plant-based meat: bridging the gap with animal meat, challenges and future prospects	Foods	Global	Plant-based meat analogs	Plant-based	Narrative review/synthesis
Lanz [51]	2024	Consumer acceptance of cultured, plant-based, 3D-printed meat and fish alternatives	Future Foods	Switzerland	Conventional meat and fish were compared with 3D-printed plant-based, cultured, 3D-printed cultured, plant-based, and 3D-printed byproduct meat and fish alternatives	Plant-based; cell-based	Quantitative
Lee [52]	2024	The role of protein blends in plant-based milk alternative: a review through the consumer lens	Trends in Food Science and Technology	Global	Plant-based milk alternatives	Plant-based	Narrative review/synthesis
Lewisch [53]	2023	Cultured meat acceptance for global food security: a systematic literature review and future research directions	Agricultural and Food Economics	Global	Cell-based meat	Cell-based	Systematic review
Lonkila [54]	2021	Promises of meat and milk alternatives: an integrative literature review on emergent research themes	Agriculture and Human Values	Global	Plant-based meat and milk, cultured meat and milk	Plant-based; cell-based	Qualitative integrative literature review
Maehle [55]	2022	Microalgae-based food: purchase intentions and willingness to pay	Future Foods	Norway	Microalgae-containing bread and beer	Algae-based	Quantitative
Malek [56]	2023	Protein source matters: understanding consumer segments with distinct preferences for alternative proteins	Future Foods	Australia	Pulses, algae, fungus (mycoprotein), whey, hemp seeds, insects, whole grains, cultured meat	Plant-based; cell-based; fungus-based; algae-based; insect-based	Quantitative
Mancini [57]	2019	European consumers' readiness to adopt insects as food. a review	Food Research International	Europe	Edible insects	Insect-based	Systematic review
Melendrez-Ruiz [58]	2019	A central place for meat, but what about pulses? Studying French consumers' representations of main dish structure, using an indirect approach	Food Research International	France	Pulses	Plant-based	Qualitative
Mellor [59]	2022	Consumer knowledge and acceptance of “algae” as a protein alternative: a UK-based qualitative study	Foods	UK	Algae and algae-based food products	Algae-based	Qualitative
Mina [60]	2023	The potential future of insects in the european food system: a systematic review based on the consumer point of view	Foods	Europe	Edible insects and insect-based processed foods	Insect-based	Systematic review
Mopendo [61]	2023	Psychosocial determinants of intentions and behavior toward edible insects in the South-Western part of the Democratic Republic of Congo	Cogent Psychology	DRC	Edible insects	Insect-based	Quantitative
Onwezen [62]	2024	A meta-review of consumer behavior studies on meat reduction and alternative protein acceptance	Food Quality and Preference	Global	Plant-based diets, meat reduction and meat replacement	All	Meta-review
Onwezen [63]	2021	A systematic review on consumer acceptance of alternative proteins: pulses, algae, insects, plant-based meat alternatives, and cultured meat	Appetite	Global	Pulses, algae, insects, plant-based alternative proteins, and cultured meat	All	Systematic review
Pakseresht [64]	2022	Review of factors affecting consumer acceptance of cultured meat	Appetite	Global	Cell-based meat	Cell-based	Systematic review
Powell [65]	2023	Perceptions and acceptance of yeast-derived dairy in British Columbia, Canada	Frontiers in Sustainable Food Systems	Canada	Yeast-derived dairy	Cell-based	Quantitative
Profeta [66]	2021	Preferences of German consumers for meat products blended with plant-based proteins	Sustainability	Germany	Composite: a food product made of 60% meat and 40% plant-based proteins	Composite	Quantitative
Profeta [67]	2021	Consumer preferences for meat blended with plant proteins – empirical findings from Belgium	Future Foods	Belgium	Composite: a food product made of 60% meat and 40% plant-based proteins	Composite	Quantitative
Puteri [68]	2023	Booming the bugs: how can marketing help increase consumer acceptance of insect-based food in Western countries?	Appetite	Western countries	Insect-based processed foods	Insect-based	Systematic review
Sato [69]	2023	Japanese attitude toward insects as food: the role of tradition	Appetite	Japan	Insects and insect-based processed foods	Insect-based	Quantitative
Siddiqui [70]	2022	Consumer acceptability of plant-, seaweed-, and insect-based foods as alternatives to meat: a critical compilation of a decade of research	Critical Reviews In Food Science And Nutrition	Global	Plant-, seaweed-, and insect-based foods	Plant-based; insect-based; Algae-based	Scoping review
Siddiqui [71]	2022	Consumer acceptance of alternative proteins: a systematic review of current alternative protein sources and interventions adapted to increase their acceptability	Sustainability	Global	Alternative proteins	All	Systematic review
Siddiqui [72]	2022	Consumer behavior toward cultured meat: a review since 2014	Appetite	Global	Cell-based meat	Cell-based	Systematic review
Śmiglak-Krajewska [73]	2021	Consumption preferences of pulses in the diet of Polish people: motives and barriers to replace animal protein with vegetable protein	Nutrients	Poland	Pulses	Plant-based	Quantitative
Sogari [74]	2023	Engaging in entomophagy: the role of food neophobia and disgust between insect and non-insect eaters	Food Quality and Preference	Belgium, China, Italy, Mexico, US	Mealworms (whole and processed products)	Insect-based	Quantitative
Spendrup [75]	2022	Consumer attitudes and beliefs toward plant-based food in different degrees of processing – the case of Sweden	Food Quality and Preference	Sweden	Plant-based meat analogs (soy mince, nuggets) and precooked beans	Plant-based	Quantitative
Szenderák [76]	2022	Consumer acceptance of plant-based meat substitutes: a narrative review	Foods	Global	Plant-based meat analogs	Plant-based	Structured narrative review
Takeda [77]	2023	Comparison of public attitudes toward 5 alternative proteins in Japan	Food Quality and Preference	Japan	Insects, plant-based meat, cultured meat, milk alternatives, and microalgae	Cell-based; plant-based; algae-based; insect-based	Quantitative
To [78]	2024	A taste of cell-cultured meat: a scoping review	Frontiers in Nutrition	Global	Cell-based meat	Cell-based	Scoping review
Tsvakirai [79]	2024	What do we know about consumers’ attitudes toward cultured meat? A scoping review	Future Foods	Global	Cell-based meat	Cell-based	Scoping review
Tzompa-Sosa [80]	2023	Consumers' acceptance toward whole and processed mealworms: A cross-country study in Belgium, China, Italy, Mexico, and the US	PLOS ONE	Belgium, China, Italy, Mexico, US	Mealworms (whole and processed products)	Insect-based	Quantitative
Tzompa-Sosa [81]	2023	What motivates consumers to accept whole and processed mealworms in their diets? A five-country study	Future Foods	Belgium, China, Italy, Mexico, US	Mealworms (whole and processed products)	Insect-based	Quantitative
Vainio [82]	2016	From beef to beans: Eating motives and the replacement of animal proteins with plant proteins among Finnish consumers	Appetite	Finland	Beans and soy products (compared with beef)	Plant-based	Quantitative
Wassman [83]	2021	Correlates of the willingness to consume insects: a meta-analysis	Journal of Insects as Food and Feed	Global	Edible insects	Insect-based	Meta-analysis following systematic review
Wassman [84]	2024	Novel microalgae-based foods: what influences Singaporean consumers’ acceptance?	Food Quality and Preference	Singapore	Microalgae	Algae-based	Quantitative
Weinrich [85]	2019	Preference and willingness to pay for meat substitutes based on microalgae	Appetite	Germany, Netherlands, France	Substitutes based on microalgae	Algae-based	Quantitative
Zhang [86]	2021	Prospects of artificial meat: opportunities and challenges around consumer acceptance	Trends in Food Science and Technology	Global	Cell-based meat; plant-based meat	Cell-based; plant-based	Narrative review/synthesis
Zollman [87]	2021	Don't have a cow, man: consumer acceptance of animal-free dairy products in 5 countries	Frontiers in Sustainable Food Systems	Brazil, Germany, India, United Kingdom, United States	Dairy products derived from precision fermentation	Cell-based	Quantitative
Zollman [88]	2023	Not getting laid: consumer acceptance of precision fermentation-made egg	Frontiers in Sustainable Food Systems	Germany, Singapore, US	Precision fermentation-made egg products	Cell-based	Quantitative

Abbreviations: 3D, three-dimenional; DRC, Democratic Republic of the Congo; UK, United Kingdom; US, United States; SSA: Sub-Saharan Africa.

### Results: General consumer acceptability of Alt-ASFs

Considering acceptance of Alt-ASFs, several central factors cut across all Alt-ASF types: sensory aspects; familiarity with the food; cooking skills to prepare the food; price; convenience (time and ease for cooking and eating); and ease of access. Sensory factors (e.g., taste, smell, and appearance) are important across all Alt-ASF types, though how this manifests differs somewhat across foods [38,43,70]. Appearance, color, and shape are the main sensory influences on preconsumption purchasing behavior and need to align with appropriate customer expectations for the product’s characteristics, whereas taste and texture matter most for shaping consumers’ views of the product postconsumption [40,50]. Familiarity and cooking skills can also be important factors, as barriers or facilitators; exposure to Alt-ASFs, particularly unfamiliar ones, generally increases acceptance [62]. Price and convenience also play some role across all Alt-ASF types [63,70].

Regarding potential health and environmental benefits of Alt-ASFs, consumers are often not well aware of these, but where they are, they can help facilitate acceptance—though results are more mixed for environmental motivations than for health ones [63]. As one review summarized, environmental and ethical considerations facilitate acceptance but not regular consumption—for which positive sensory attributes are necessary [54]. In general, tangible perceived personal benefits related to health, safety, nutrition, and taste are more powerful than broader societal ones in terms of increasing consumers’ willingness to adopt Alt-ASFs [62]. Situational factors also play a role, with meat seen as more central to situations like special occasions, business lunches, “Sunday dinners,” and similar, lowering Alt-ASF acceptability within those contexts [38,76]. Finally, some consumers view Alt-ASFs as a separate category from ASFs, as opposed to a substitute, making them willing to consume them but not necessarily as ASF replacements—which may entail limited effects on the environmental sustainability of the food system [43].

Importantly, for many consumers, the barriers to Alt-ASF consumption may be stronger than the facilitators. A trend consistent across several reviewed studies was that, although consumers may perceive some benefits of Alt-ASFs to society (e.g., environmental sustainability, animal welfare), they see fewer benefits accruing to them personally and, in the case of insect- and cell-based foods, potentially more personal risk [32,64]. As one study noted, reflecting a common finding, “Although consumers consider sustainability to be very important, immediate benefits for the self are usually more important than long-term benefits for the community in everyday decisions related to consumer choices” [60]. More personal motivations (like concerns for one’s health, connected to nutrition or food safety concerns associated with ASFs) may be needed to motivate dietary shifts toward Alt-ASFs for many consumers.

Acceptance is about not only a food’s characteristics but also how those are interpreted compared with expectations. For the Alt-ASFs that position themselves most clearly as replacements to ASFs (e.g., soy milk for dairy milk; plant-based meat analogs for meat), the consumer is primed to make a comparison to that ASF. This creates a set of expectations against which the Alt-ASF is compared, which may be difficult to meet in some cases (e.g., sensory aspects) [43]. In contrast, Alt-ASFs that are not as clearly positioned as direct replacements (e.g., chickpeas, insect snacks) may be evaluated more on their own characteristics or in comparison to other known foods that the consumer places in the same dietary category (e.g., comparing insect snacks to potato crisps) [77].

The reviewed research consistently showed variations in Alt-ASF acceptability by sex, with two-thirds of included reviews reporting sex differences: women were generally found to be more willing than men to reduce ASF consumption for ethical or environmental reasons, perhaps because of the importance of meat in many cultural definitions of masculinity in the countries where the topic was studied [64]. Women were also often found to be more accepting of plant-based milk and perhaps other plant-based products. However, some studies found that women were significantly less accepting than men of cell-based meat and insect-based foods, with both results being strongly consistent across studies and contexts. However, the narrow geographical scope of the studies limits the extrapolation of these findings.

## Results: How Acceptability Varies by Alt-ASF Type

Comparisons of acceptability across different Alt-ASFs from diverse contexts generally find that plant-based products are more acceptable than cell-based products, which are more acceptable than insect-based products [32,60,63,76,77,78]. There are insufficient comparative data to clearly rank algae-based and fungus-based products among these, but algae-based products may be more acceptable in Asian countries that have a strong tradition of algae consumption [77]. The following subsections summarize the review results for each type of Alt-ASF.

### Plant-based foods

Starting at the “most acceptable” end of the Alt-ASF acceptability spectrum, plant-based foods are fairly widely accepted as foods that people would be willing to try or eat occasionally [63,76,86]. This is particularly true for plant-based milk alternatives, which have gained a significant market share in some HICs and across the income spectrum in parts of East/Southeast Asia; for example, in the United States, 40% of households purchased plant-based milk in 2024 [89]. However, consumers often have lower willingness to consume plant-based Alt-ASFs regularly or use them to replace ASFs, largely due to sensory factors: perceived poor taste, texture, smell, and appearance when compared with ASFs [29,50]. Highly processed plant-based meat and dairy alternatives were seen as easy to prepare and eat, whereas for minimally processed legumes, inconvenience and a lack of cooking skills were important barriers [44,73,82]. There was limited research on traditional plant-based processed products, like tofu and tempeh, although some reviews noted these as having limited sensory appeal to most Western consumers, due to differences with traditional ASFs; in the United States, for example, tofu, tempeh, and seitan are consumed in about half as many households as plant-based meat, despite being longstanding products [40,70,89].

### Fungus-based foods

The evidence on fungus-based foods was limited. Only 2 studies focused on these foods, and 2 included them as one specific category among multiple examined; several additional studies seemed to implicitly group them with plant-based meat analogs. A few studies were specific to mycoproteins and present a similar picture to the highly processed plant-based foods: there was limited outright consumer rejection or serious concern or dislike, but poor sensory characteristics, price, and perceived ambiguous nutritional value posed barriers to sustained consumption [35,36]. The only potential difference was some mention of food neophobia as a barrier for mycoproteins, whereas it was not mentioned for plant-based meat analogs [35,37]. There is generally low consumer awareness of mycoprotein foods specifically, with little evidence that consumers clearly distinguish between mycoproteins and plant-based meat analogs when it comes to acceptability [90]. These products usually appear side by side on store shelves without clear labeling differences; many are blended with other ingredients, making them difficult for consumers to distinguish [38].

### Algae-based foods

The reviewed research suggests that algae-based foods are fairly well accepted in the limited locations studied, being viewed as healthy, natural, and environmentally sustainable, although also with barriers related to taste, cost, and awareness [45,59,63,70,77,84]. Algae-based foods, however, come with an important caveat: it is unclear whether consumers view them as substitutes for ASFs and would consume them in that way [59,84]. Currently, algae-based foods rarely appear in place of ASFs in diets, and most products on the market are not marketed as ASF alternatives [39,59]. Instead, macroalgae are usually positioned as an ingredient in a dish that includes ASF or another Alt-ASF (e.g., sushi, secondary to fish; ramen, garnishing a dish with meat or tofu), a snack (algae sheets or crackers), or a vegetable-like side dish. Microalgae mostly appear as a supplement or ingredient in processed foods such as cookies, beverages, bread, and crackers; whereas some meat–microalgae composite foods have been developed, to date these usually include only very small amounts of microalgae [91]. Some ambiguity in terms of whether Alt-ASFs are actually viewed as Alt-ASFs also exists for minimally processed plant-based foods (e.g., nuts, seeds) and insects (e.g., when sold in processed snacks), but it seems to be greatest for algae. This is an important focus for future research on algae-based foods and a factor to consider in policy recommendations.

### Cell-based foods

For cell-based foods, the literature is hypothetical, sensitive to framing effects, and exclusive to HIC contexts. One may expect results to change once (if) such foods become widely available; at present, however, results for acceptability are inconsistent. Particularly when discounting industry-sponsored studies (where authors had potential conflicts of interest), results suggest consumer hesitancy and several strong barriers to consumption, at least for cell-based meat [31,32,54,63,64,72,78]. These include feelings of disgust, perceiving the product as unnatural, food safety worries (particularly about long-term unknown effects), and (to a lesser extent) concerns related to industry power and distrust of science or technology [32,48,64]. Facilitating factors (in addition to those discussed above as cross-cutting, such as potential environmental and health benefits) include perceived potential food safety and animal welfare benefits [31,48,72]. The connection to animal welfare seems to be strongest in consumers’ minds for cell-based meat, perhaps due to what many feel to be a clear resemblance to meat but without (or with very limited) direct harm to animals. Although evidence is more limited, it suggests that precision-fermented egg and especially dairy products may be more accepted than cell-based meat, with more limited feelings of disgust and worries about unnaturalness and safety [47,65,87,88].

### Insect-based foods

The largest barriers to Alt-ASF acceptance exist for insect-based foods in societies that have not traditionally consumed insects. Feelings of disgust, food neophobia, perceived health or food safety risks (e.g., chemical contamination, toxins, and microbes), social norms, and poor taste expectations were all found in reviewed studies to be serious barriers to consumption, as were more situational factors like a lack of access and not knowing how to prepare them [28,41,43,49,57,60,63,71,83]. The traditional food choice drivers, such as taste, affordability, and convenience, were dominated by more fundamental concerns over whether insects were, in fact, a safe food to eat—or even a food at all [60,28,41,49,57,71,83]. Among facilitating factors, familiarity and curiosity played a role, as did the inclusion of “invisible” insect ingredients in well-known foods [28,41,43,49,57]. In cultures with traditions of insect consumption, which are generally non-Western, acceptability is higher and insect-based foods may be widely appreciated—and even highly valued, prestige foods [28,41,61,[80], [81], [82], [83], [84], [85], [86], [87], [88], [89], [90], [91], [92]]. However, not all non-Western countries have such traditions of insect consumption, and even in some that do, there may also be negative associations with insect-based foods (e.g., as being old-fashioned as opposed to modern) [33,34,69].

### Composite foods

The limited evidence on composite foods (those mixing Alt-ASFs and ASFs into one food item) has focused on plant-based ingredients combined with meat [40]. It suggests that such products face similar barriers and facilitators (poorer sensory qualities but seen as healthier) but perhaps with lower barriers, particularly as consumers may heuristically categorize them as meat and thus may not require as large a mental shift to adopt them.

## Discussion

This paper has reviewed the literature on consumer acceptability of Alt-ASFs, considering a wide range of Alt-ASF types, with particular focus on the differences among them. On the first research question, on the state of research on Alt-ASF acceptability, we find it to be a highly active topic of research, particularly for insect- and cell-based foods and plant-based meat analogs in certain HICs. Nonetheless, there remain gaps. Algae-based foods remain underexplored; additional research would be helpful, particularly probing the extent to which consumers are ready to use these foods as replacements for ASFs. Composite foods are also less studied; more work in the future could focus on different types of composites (e.g., including fungus- or cell-based ingredients; considering dairy composites) and how well they meet consumer preferences while improving on certain aspects of ASFs. Geographically, more research in LMICs on foods other than insects is needed to understand the acceptability of Alt-ASFs in these contexts. Regionally, the Middle East and North Africa and Central Asia have limited research on all Alt-ASF types, whereas Sub-Saharan Africa has limited research on Alt-ASFs other than insects. Addressing these gaps will be important for ensuring that research can equitably inform a just transition to sustainable healthy diets. Methodologically, more research using qualitative methods, less reliance on paid online survey panels (which may have weaknesses in quality and representativeness), and more studying regular consumption as opposed to “willingness to try” would be valuable.

**FIGURE 4:**
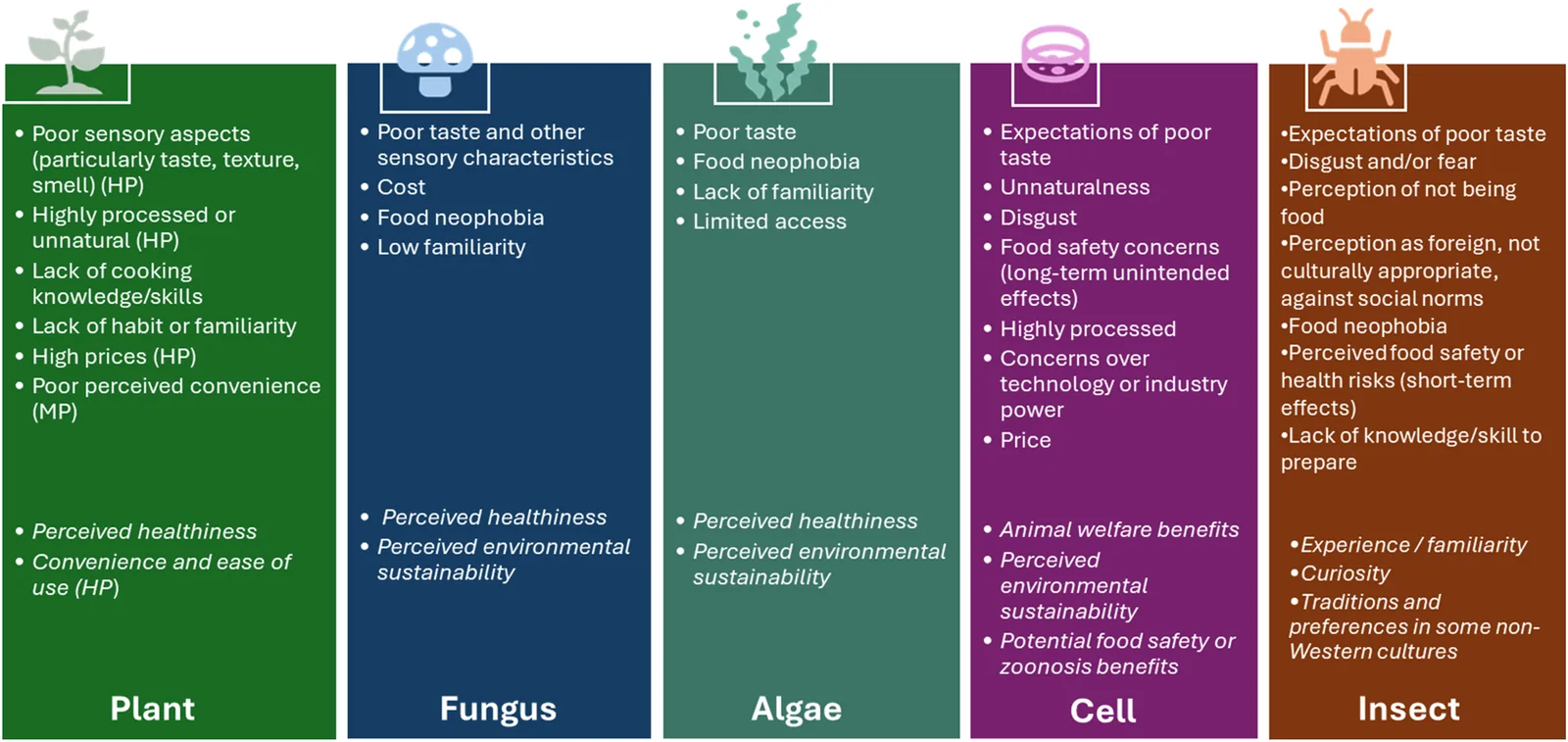
Acceptability of different Alt-ASF: main results of the review. Text in italics at the bottom of each rectangle indicates particular facilitators to that Alt-ASF’s consumption, whereas the nonitalic text at the top of the rectangle indicates particular barriers to that Alt-ASF’s consumption. For plant-based foods, “HP” indicates results specific to highly processed foods (meat and dairy analogs), whereas “MP” indicates results specific to the minimally processed foods. There were limited data for fungus-based foods, specifically. Alt-ASF, alternative to animal-source food.

Regarding the second research question, on the acceptability of different types of Alt-ASFs, we find that many Alt-ASFs face barriers when it comes to consumer acceptability, but that there are clear differences across the Alt-ASF types. Figure 4 summarizes the review’s results by Alt-ASF category. It places Alt-ASFs on a spectrum of acceptability, from plant-based as most acceptable (left-hand side) to insect-based as least acceptable (right-hand side).

Importantly, food acceptability should not be seen as a static or passive concept: it is shaped by consumers, food companies’ products and advertising, and policy. It changes over time at the individual and societal levels. Such change is already apparent for Alt-ASFs in some markets: in the United States, for example, the market for plant-based foods grew from $3.9 billion in 2017 to $8.1 billion in 2023—though unit sales declined from 2021–2023 [93]. The number of companies engaged in producing fermented and cultivated Alt-ASFs has also grown in recent years, with projections for significant sales growth in the future [90,94]. Most world cultures are characterized by large historic changes in diets and in what is seen as traditional, central foods, with these changes having advantages and drawbacks. For example, although legumes may not be widely consumed in many HICs at present, they were once much more common; in France, per-capita legume consumption has fallen by 76% in the past century [58]. At the same time, globalization and increased exposure to ‘foreign’ foods have made many Western consumers accustomed to soy-based Alt-ASFs such as tofu. Even unintentional changes in food culture can thus result in significant shifts in Alt-ASF acceptance, and intentional efforts can likely bring about similar or larger changes.

Across multiple types of Alt-ASFs, research has found that some consumers, particularly high-income Western consumers, are wary of highly processed foods. This emerged as a barrier to acceptance in multiple studies across algae-, cell-, fungus-, and plant-based foods [33,39,56,64,65] and may be related to media coverage of ultraprocessed foods in recent years [95]. The reliance on extensive processing may be difficult to change for many of these foods, as it is inherent to the foods’ ability to mimic sensory characteristics of ASFs. This highlights a key tension: meeting multiple consumer needs simultaneously can be challenging and require trade-offs among aspects of acceptability or between acceptability and other desired product attributes. For example, although legumes are often associated with requiring more cooking effort to become appealing, plant-based meat analogs are convenient and somewhat resemble meats, mitigating these barriers—but those characteristics rely on a high degree of processing and added ingredients, which may be seen by consumers as unhealthy or unnatural. Checking all the “boxes” for acceptable, affordable, nutritious, safe, and environmentally sustainable Alt-ASFs is thus not a simple project.

Several reviewed studies note that labeling, including any requirements, may affect acceptability of Alt-ASFs. This includes marketing using similar terms to ASFs, such as being able to refer to plant-based milks as “milk” as opposed to “nondairy drinks” or similar; the use of ASF-like terms is thought to promote consumer acceptance but can also imply equivalence that does not exist and is banned or discouraged in some countries [93]. Whether labels can include claims related to health, environmental, or animal welfare benefits is also a relevant topic, as these factors have been identified as drivers of consumers choosing Alt-ASF products [38,43,62]. However, some regulations restrict this; for example, regulations proposed in Brazil in 2023 suggested requiring a notice on plant-based meat alternatives that they do *not* replace their animal equivalents in nutritional or functional terms [93]. Such regulations, usually intended to protect consumers or industry, have implications for consumer acceptability.

As a new product, cultured meat offers a particularly strong case in point. Consumers have been shown to be sensitive to different uses of terminology, with a general preference for the word “cultivated” as opposed to “cell-cultured” or “lab-grown,” and the terms used in labeling will likely influence acceptance [94]. No countries have adopted comprehensive rules for labeling cultured meat, although Singapore (the most advanced market for cultivated meat) has indicated that such products must be clearly signaled as being cultivated or cell-based. However, some United States states have proposed or enacted laws that restrict cultivated meat from being labeled as meat or using related terms such as “burger” or “sausage” or require inclusion of words such as “lab-grown” [94]. Consumers have also been shown to be motivated by marketing messages that emphasize the cleanliness and safety of cultivated meat, particularly where it is claimed to be “cleaner” than animal meat [94]. It is not yet clear whether companies will be able to make such claims, nor is it clear whether cultivated meat can be considered and labeled as being vegan, which will be critical for appealing to that demographic [65].

### Limitations of the review

There are several limitations to this review. First, the search was limited to English-language publications, and thus may have excluded some non-English resources—particularly those focused on Latin America. Moreover, it used only used 2 databases, although database-related bias was mitigated by using Scopus, the largest single academic database, which spans many disciplines. The focus of the search on abstracts, titles, and keywords may have excluded some sources that discussed acceptability as only one topic among many, not as a main focus. Google Scholar also has the weakness of irreproducibility: due to a lack of transparent and stable acronyms, a future search using the same approach could return different results. No gray literature sources were included, no snowball searching was undertaken, and, due to feasibility, only less than one-quarter of identified scientific papers that passed the abstract-screening stage were included in the full-text review, meaning it does not reflect all eligible studies identified during screening. Reviewer discretion in the criteria used to select which studies to include could have introduced bias. Although a broad set of Alt-ASFs were included, we did not specifically include “yeast” among our search terms, even though it is part of the fungi kingdom and can be a food source. The review is thus far from comprehensive. Finally, only one reviewer was involved in screening and data extraction, which may have introduced some bias to the results. This was mitigated by having clearly defined inclusion and exclusion criteria, multiple researchers reviewing and approving the search strategy, multiple reviewers of the draft report, and a default decision of inclusion where there was ambiguity between inclusion and exclusion criteria.

### Policy and programmatic implications

Increasing consumption of Alt-ASF will require both supply- and demand-side shifts. On the supply side, many current Alt-ASFs do not meet consumer expectations, particularly when it comes to sensory aspects that align with the ASF eating experience. It is thus important to invest in improving products’ appearance, taste, and texture, at least for products that are clearly positioned as analogs to traditional ASFs (e.g., burgers, sausages). For legumes and other non-ASF analogs (e.g., tofu), convenience improvements are also important. Across all Alt-ASF types, price is essential: many consumers expect Alt-ASFs to be at parity with or cheaper than comparable ASFs, whereas most are considerably more expensive [21]. Lowering costs is thus an essential dimension of future product development if Alt-ASFs are to be accepted by a mass market.

On the demand side, many consumers have limited awareness of Alt-ASFs and their potential advantages and drawbacks, how they compare to one another, and why one might choose to consume them in place of ASFs (or not). Although the literature is clear that, for most consumers, such awareness cannot overcome more practical or personal barriers (e.g., high prices, food safety fears), it can play some role in fostering adoption. Importantly, a transition to more environmentally sustainable diets with fewer ASFs depends not only on Alt-ASF acceptability but also on consumer willingness to reduce ASF consumption. This is not implicit in the acceptability of Alt-ASFs, as Alt-ASFs could also be consumed in addition to ASFs. Willingness to reduce ASF consumption has generally been found to be low, although it varies across cultures and demographic groups [38,43,96]. Attachment to ASFs is driven by many factors, including sensory pleasure, a symbolic cultural place (e.g., signaling high social status), habit (e.g., seeing meat as synonymous with a meal), associations with health and material wellbeing, and associations with masculinity [38,62]. Although environmental factors can be motivators for dietary change [70], there is limited consumer awareness of the environmental impacts of meat production [43]. However, few consumers stop eating meat primarily for environmental reasons, which are typically peripheral and may not outweigh sensory and price factors [29,62,70,76]. Indeed, some consumers may be actively resistant to information about environmental or animal welfare aspects of ASF production [62].

Efforts to increase awareness and motivation are thus needed, focusing not only on the Alt-ASFs themselves but also on consuming them in place of high-impact and less nutritious ASFs, like processed meat. Marketing campaigns for sustainable, healthy diets or specific Alt-ASF types, targeted to specific consumer groups, may be able to play a role in increasing acceptability, although evidence of their effectiveness for promoting healthy or environmentally sustainable eating is mixed [97,98]. In particular, information provision alone is unlikely to alter the choices of those who are truly resistant to Alt-ASFs or to reducing meat consumption [96]. Labeling and consistency in terminology are necessary enablers to support consumer choice, as even a motivated and willing consumer may struggle to choose appropriate Alt-ASFs if they are not able to clearly identify them at the point of purchase. Ideal labeling to support consumer choice would allow for describing Alt-ASFs in ASF-like terms (e.g., “milk”) and flagging Alt-ASFs’ nutritional and environmental sustainability benefits, where robustly verified [99]. Finally, for some Alt-ASFs (e.g., insects, legumes), limited cooking skills can also serve as barriers, suggesting a role for skill-based consumer education efforts (e.g., free recipes, cooking demonstrations at retail or food service locations) and development of convenient, easy-to-prepare products.

Where efforts at shifting Alt-ASF supply and demand are delivered using public funding, they should focus on Alt-ASFs with positive profiles for nutritional content and environmental sustainability, as these can vary widely by Alt-ASF type [18,19,99]—and on encouraging these to be consumed in place of the highest-impact ASFs. Strategically, they should also focus on those foods for which acceptability is most achievable; the results of the review suggest that these are primarily plant- and fungus-based foods, with barriers being considerably higher for insect-based foods in areas where they are not traditionally consumed and for cell-based meat. Although algae-based foods face moderate acceptability barriers, they are also not widely seen as an ASF replacement, suggesting that significantly reduced ASF consumption is unlikely to be a consequence of increased algae-based food consumption. Focusing on those consumers in the middle of the spectrum—neither early adopters of Alt-ASFs nor those most resistant to adopting them—may also be a strategic choice [96].

In conclusion, global food system is currently environmentally unsustainable, contributing to greenhouse gas emissions, water pollution, reductions in biodiversity, and other challenges [13]. Although ASFs provide essential nutrients in dense, readily absorbable forms, ASF production is a large contributor to these burdens, and certain ASFs are associated with negative health effects, antibiotic resistance, and animal welfare issues. Replacing some ASFs in the diet with Alt-ASFs, particularly for high-consuming populations, offers a potential solution as part of a wider transition to sustainable food systems. However, such a transition will only happen at an impactful scale if Alt-ASF products are widely accepted and adopted by consumers. This paper reviewed the literature on consumer acceptability of Alt-ASFs, finding that acceptability generally remains limited but tends to be highest for plant-based foods. Barriers to Alt-ASF acceptability range from the readily modifiable—unfamiliarity, high relative prices, and limited cooking skills—to the much more challenging—fear and disgust. Acceptability varies considerably by type of Alt-ASF, as well as by context; strategies to encourage increased Alt-ASF consumption as part of an equitable transition to sustainable food systems must thus consider the acceptability of specific Alt-ASFs, to specific consumer groups, in specific contexts.

## Author contributions

The authors’ responsibilities were as follows – SN: conceptualization; methodology; literature search, screening, and data extraction; wrote the original draft; LMN: conceptualization, funding, and reviewed and edited the original draft. TB: methodology and reviewed and edited the original draft. MH, MX, KS: reviewed and edited the original draft; and all authors: read and approved the final version.

## Data availability

Data described in the manuscript, codebook, and analytic code will be made available upon request pending.

## Declaration of generative AI and AI-assisted technologies in the writing process

The authors declare that no generative AI or AI-assisted technologies were used in the writing of this manuscript.

## Funding

This work was commissioned and funded by the Food and Agriculture Organization of the United Nations through a grant to GAIN (PO 364309). The views expressed in this publication are those of the author(s) and do not necessarily reflect the views or policies of the Food and Agriculture Organization of the United Nations.

## Conflict of interest

The authors report no conflicts of interest.

## References

[1] Beal T., Gardner C.D., Herrero M., Iannotti L.L., Merbold L., Nordhagen S. (2023). Friend or Foe? The role of animal-source foods in healthy and environmentally sustainable diets. J. Nutr..

[2] Nordhagen S. (2026). Animal-source foods for nutrition, environment and society: finding a balance. Proc. Nutr. Soc..

[3] Willett W., Rockström J., Loken B., Springmann M., Lang T., Vermeulen S. (2019). Food in the Anthropocene: the EAT–Lancet Commission on healthy diets from sustainable food systems. Lancet..

[4] FAO (2009).

[5] Poore J., Nemecek T. (2018). Reducing food’s environmental impacts through producers and consumers. Science.

[6] UNEP (2023).

[7] Gerber P., Steinfeld H., Henderson B., Mottet A., Opio C., Dijkman J. (2013).

[8] Godfray H.C.J., Aveyard P., Garnett T., Hall J.W., Key T.J., Lorimer J. (2018). Meat consumption, health, and the environment. Science.

[9] Barange M., Bahri T., Beveridge M., Cochrane K., Funge-Smith S., Poulain F. (2018).

[10] Foster G., Rahmstorf S. (2026). Global warming has accelerated significantly. Geophys. Res. Lett..

[11] Pelletier N., Tyedmers P. (2010). Forecasting potential global environmental costs of livestock production 2000–2050. Proc. Natl. Acad. Sci. U. S. A..

[12] FAOSTAT (2018). http://www.fao.org/faostat/en/#data/FBS.

[13] Rockström J., Thilsted S.H., Willett W.C., Gordon L.J., Herrero M., Hicks C.C. (2025). The EAT–Lancet Commission on healthy, sustainable, and just food systems. Lancet.

[14] Hale G., Oncescu V., Bhangale R. (2025). Pace of adoption of alternatives to animal-source foods is an important factor in reaching climate goals. Sci. Rep..

[15] Hawkes C., Ruel M.T. (2020). New Delhi, India; 2011 (Prepared for the IFPRI 2020 international conference “Leveraging Agriculture for Improving Nutrition and Health).

[16] House J., Brons A., Wertheim-Heck S., Van Der Horst H. (2024). What is culturally appropriate food consumption? A systematic literature review exploring six conceptual themes and their implications for sustainable food system transformation. Agric. Hum. Values..

[17] Burlingame B., Dernini S. (2012).

[18] Beal T., McLaren R., Saxena T., Sokourenko K., Nordhagen S., Lambertini E. (2026). Nutritional and health impacts of alternative animal source foods: a scoping review. Lancet Planet. Health.

[19] Sokourenko K., Beal T., McAuliffe G., Young O., Gould Z., Quint B. (2026). Environmental impacts of alternatives to animal-source foods: a scoping review. Lancet Planet. Health.

[20] Nordhagen S., Beal T., Herrero M., Mane E., Neufeld L.M., Sokourenko K. (2026). Livelihood implications of dietary shifts to animal-source food alternatives: a scoping review. Lancet Planet. Health.

[21] Nordhagen S., Beal T., Herrero M., Neufeld L.M., Sokourenko K. (2026). Prices and cost drivers of alternatives to animal-source foods: a scoping review and narrative analysis. Lancet Planet. Health.

[22] Bedsaul-Fryer J.R., Monroy-Gomez J., Van Zutphen-Küffer K.G., Kraemer K. (2024). An introduction to traditional and novel alternative proteins for low- and middle-income countries. Curr. Dev. Nutr..

[23] Arksey H., O’Malley L. (2005). Scoping studies: towards a methodological framework. Int. J. Soc. Res. Methodol..

[24] Levac D., Colquhoun H., O’Brien K.K. (2010). Scoping studies: advancing the methodology, Implement. Sci..

[25] Tricco A.C., Lillie E., Zarin W., O’Brien K.K., Colquhoun H., Levac D. (2018). PRISMA Extension for Scoping Reviews (PRISMA-ScR): checklist and explanation. Ann. Intern. Med..

[26] Pellack L. (2026). https://instr.iastate.libguides.com/c.php?g=901522&p=6492159.

[27] Page M.J., McKenzie J.E., Bossuyt P.M., Boutron I., Hoffmann T.C., Mulrow C.D. (2021). The PRISMA 2020 statement: an updated guideline for reporting systematic reviews. BMJ.

[28] Alhujaili A., Nocella G., Macready A. (2023). Insects as food: consumers’ acceptance and marketing. Foods.

[29] Appiani M., Cattaneo C., Laureati M. (2023). Sensory properties and consumer acceptance of plant-based meat, dairy, fish and eggs analogs: a systematic review. Front. Sustain. Food Syst..

[30] Banovic M., Barone A.M., Asioli D., Grasso S. (2022). Enabling sustainable plant-forward transition: European consumer attitudes and intention to buy hybrid products. Food Qual. Prefer..

[31] Bryant C., Barnett J. (2018). Consumer acceptance of cultured meat: a systematic review. Meat Sci.

[32] Bryant C., Barnett J. (2020). Consumer acceptance of cultured meat: an updated review (2018–2020). Appl. Sci..

[33] Chia A., Shou Y., Wong N.M.Y., Cameron-Smith D., Sim X., Van Dam R.M. (2024). Complexity of consumer acceptance to alternative protein foods in a multiethnic Asian population: a comparison of plant-based meat alternatives, cultured meat, and insect-based products. Food Qual. Prefer..

[34] da Cunha C.F., da Silva M.B. de O., Cheung T.L. (2022). Understanding the perception of edible insects. Br. Food J..

[35] De Cianni R., Pippinato L., Mancuso T. (2023). A systematic review on drivers influencing consumption of edible mushrooms and innovative mushroom-containing products. Appetite.

[36] Dean D., Rombach M., de Koning W., Vriesekoop F., Satyajaya W., Yuliandari P. (2022). Understanding key factors influencing consumers’ willingness to try, buy, and pay a price premium for mycoproteins. Nutrients.

[37] Dean D., Rombach M., Vriesekoop F., de Koning W., Aguiar L.K., Anderson M. (2023). Should I really pay a premium for this? Consumer perspectives on cultured muscle, plant-based and fungal-based protein as meat alternatives. J. Int. Food Agribus. Mark..

[38] Eckl M.R., Biesbroek S., Van’t Veer P., Geleijnse J.M. (2021). Replacement of meat with non-meat protein sources: a review of the drivers and inhibitors in developed countries. Nutrients.

[39] Embling R., Neilson L., Randall T., Mellor C., Lee M.D., Wilkinson L.L. (2022). ‘Edible seaweeds’ as an alternative to animal-based proteins in the UK: Identifying product beliefs and consumer traits as drivers of consumer acceptability for macroalgae. Food Qual. Prefer..

[40] Fiorentini M., Kinchla A.J., Nolden A.A. (2020). Role of sensory evaluation in consumer acceptance of plant-based meat analogs and meat extenders: a scoping review. Foods.

[41] Florença S.G., Guiné R.P.F., Gonçalves F.J.A., Barroca M.J., Ferreira M., Costa C.A. (2022). The motivations for consumption of edible insects: a systematic review. Foods.

[42] Gbejewoh O., Marais J., Erasmus S.W. (2022). Planetary health and the promises of plant-based meat from a sub-Saharan African perspective: a review. Sci. Afr..

[43] Hartmann C., Siegrist M. (2017). Consumer perception and behaviour regarding sustainable protein consumption: a systematic review. Trends Food Sci. Technol..

[44] Henn K., Bøye Olsen S.B., Goddyn H., Bredie W.L.P. (2022). Willingness to replace animal-based products with pulses among consumers in different European countries. Food Res. Int..

[45] Islam M.S., Kabir K.M.A., Islam Md.S., Saha B.B. (2023). The perception of consumers towards microalgae as an alternative food resource in Bangladesh: a contingent valuation approach. Evergreen.

[46] Jallinoja P., Niva M., Latvala T. (2016). Future of sustainable eating? Examining the potential for expanding bean eating in a meat-eating culture. Futures.

[47] Klöckner C.A., Engel L., Moritz J., Burton R.J., Young J.F., Kidmose U. (2022). Milk, meat, and fish from the petri dish—which attributes would make cultured proteins (un)attractive and for whom? Results from a Nordic Survey. Front. Sustain. Food Syst..

[48] Kouarfaté B.B., Durif F.N. (2023). A systematic review of determinants of cultured meat adoption: impacts and guiding insights. Br. Food J..

[49] Kröger T., Dupont J., Büsing L., Fiebelkorn F. (2022). Acceptance of insect-based food products in Western Societies: a systematic review. Front. Nutr..

[50] Kumari S., Alam A.N., Hossain M.J., Lee E.Y., Hwang Y.H., Joo S.T. (2024). Sensory evaluation of plant-based meat: bridging the gap with animal meat, challenges and future prospects. Foods.

[51] Lanz M., Hartmann C., Egan P., Siegrist M. (2024). Consumer acceptance of cultured, plant-based, 3D-printed meat and fish alternatives. Future Foods.

[52] Lee P.Y., Leong S.Y., Oey I. (2024). The role of protein blends in plant-based milk alternative: a review through the consumer lens. Trends Food Sci. Technol..

[53] Lewisch L., Riefler P. (2023). Cultured meat acceptance for global food security: a systematic literature review and future research directions. Agric. Food Econ..

[54] Lonkila A., Kaljonen M. (2021). Promises of meat and milk alternatives: an integrative literature review on emergent research themes. Agric. Hum. Values..

[55] Maehle N., Skjeret F. (2022). Microalgae-based food: purchase intentions and willingness to pay. Future Foods.

[56] Malek L., Umberger W. (2023). Protein source matters: understanding consumer segments with distinct preferences for alternative proteins. Future Foods.

[57] Mancini S., Moruzzo R., Riccioli F., Paci G. (2019). European consumers’ readiness to adopt insects as food. A review. Food Res. Int..

[58] Melendrez-Ruiz J., Chambaron S., Buatois Q., Monnery-Patris S., Arvisenet G. (2019). A central place for meat, but what about pulses? Studying French consumers’ representations of main dish structure, using an indirect approach. Food Res. Int..

[59] Mellor C., Embling R., Neilson L., Randall T., Wakeham C., Lee M.D. (2022). Consumer knowledge and acceptance of ‘Algae’ as a protein alternative: a UK-based qualitative study. Foods.

[60] Mina G., Peira G., Bonadonna A. (2023). The potential future of insects in the European Food System: a systematic review based on the consumer point of view. Foods.

[61] Mopendo Mwisomi E., Luminet O., Chang B., Manwanina Kiumba N., Schmitz M. (2023). Psychosocial determinants of intentions and behaviour towards edible insects in the South-Western part of the Democratic Republic of Congo, Cogent. Psychol.

[62] Onwezen M.C., Dagevos H. (2024). A meta-review of consumer behaviour studies on meat reduction and alternative protein acceptance. Food Qual. Prefer..

[63] Onwezen M.C., Bouwman E.P., Reinders M.J., Dagevos H. (2021). A systematic review on consumer acceptance of alternative proteins: pulses, algae, insects, plant-based meat alternatives, and cultured meat. Appetite.

[64] Pakseresht A., Ahmadi Kaliji S., Canavari M. (2022). Review of factors affecting consumer acceptance of cultured meat. Appetite.

[65] Powell L.J., Mendly-Zambo Z., Newman L.L. (2023). Perceptions and acceptance of yeast-derived dairy in British Columbia, Canada. Front. Sustain. Food Syst..

[66] Profeta A., Baune M.C., Smetana S., Bornkessel S., Broucke K., Van Royen G. (2021). Preferences of German consumers for meat products blended with plant-based proteins. Sustainability.

[67] Profeta A., Baune M.C., Smetana S., Broucke K., Van Royen G., Weiss J. (2021). Consumer preferences for meat blended with plant proteins—empirical findings from Belgium. Future Foods.

[68] Puteri B., Jahnke B., Zander K. (2023). Booming the bugs: how can marketing help increase consumer acceptance of insect-based food in Western countries?. Appetite.

[69] Sato K., Ishizuka N. (2023). Japanese attitude toward insects as food: the role of tradition. Appetite.

[70] Siddiqui S.A., Bahmid N.A., Mahmud C.M.M., Boukid F., Lamri M., Gagaoua M. (2023). Consumer acceptability of plant-, seaweed-, and insect-based foods as alternatives to meat: a critical compilation of a decade of research. Crit. Rev. Food Sci. Nutr..

[71] Siddiqui S.A., Alvi T., Sameen A., Khan S., Blinov A.V., Nagdalian A.A. (2022). Consumer acceptance of alternative proteins: a systematic review of current alternative protein sources and interventions adapted to increase their acceptability. Sustainability.

[72] Siddiqui S.A., Khan S., Ullah Farooqi M.Q., Singh P., Fernando I., Nagdalian A. (2022). Consumer behavior towards cultured meat: a review since 2014. Appetite.

[73] Śmiglak-Krajewska M., Wojciechowska-Solis J. (2021). Consumption preferences of pulses in the diet of Polish people: motives and barriers to replace animal protein with vegetable protein. Nutrients.

[74] Sogari G., Riccioli F., Moruzzo R., Menozzi D., Tzompa Sosa D.A., Li J. (2023). Engaging in entomophagy: the role of food neophobia and disgust between insect and non-insect eaters. Food Qual. Prefer..

[75] Spendrup S., Hovmalm H.P. (2022). Consumer attitudes and beliefs towards plant-based food in different degrees of processing—the case of Sweden. Food Qual. Prefer..

[76] Szenderák J., Fróna D., Rákos M. (2022). Consumer acceptance of plant-based meat substitutes: a narrative review. Foods.

[77] Takeda K.F., Yazawa A., Yamaguchi Y., Koizumi N., Shineha R. (2023). Comparison of public attitudes toward five alternative proteins in Japan. Food Qual. Prefer..

[78] To K.V., Comer C.C., O’Keefe S.F., Lahne J. (2024). A taste of cell-cultured meat: a scoping review. Front. Nutr..

[79] Tsvakirai C.Z., Nalley L.L., Tshehla M. (2024). What do we know about consumers’ attitudes towards cultured meat? A scoping review. Future Foods.

[80] Tzompa-Sosa D.A., Moruzzo R., Mancini S., Schouteten J.J., Liu A., Li J. (2023). Consumers’ acceptance toward whole and processed mealworms: a cross-country study in Belgium, China, Italy, Mexico, and the US. PLOS ONE.

[81] Tzompa-Sosa D.A., Sogari G., Copelotti E., Andreani G., Schouteten J.J., Moruzzo R. (2023). What motivates consumers to accept whole and processed mealworms in their diets? A five-country study. Future Foods.

[82] Vainio A., Niva M., Jallinoja P., Latvala T. (2016). From beef to beans: eating motives and the replacement of animal proteins with plant proteins among Finnish consumers. Appetite.

[83] Wassmann B., Siegrist M., Hartmann C. (2021). Correlates of the willingness to consume insects: a meta-analysis. J. Insects Food Feed..

[84] Wassmann B., Hartmann C., Siegrist M. (2024). Novel microalgae-based foods: what influences Singaporean consumers’ acceptance?. Food Qual. Prefer..

[85] Weinrich R., Elshiewy O. (2019). Preference and willingness to pay for meat substitutes based on micro-algae. Appetite.

[86] Zhang L., Hu Y., Badar I.H., Xia X., Kong B., Chen Q. (2021). Prospects of artificial meat: opportunities and challenges around consumer acceptance. Trends Food Sci. Technol..

[87] Zollman Thomas O., Bryant C. (2021). Don’t have a cow, man: consumer acceptance of animal-free dairy products in five countries. Front. Sustain. Food Syst..

[88] Zollman Thomas O., Chong M., Leung A., Fernandez T.M., Ng S.T. (2023). Not getting laid: consumer acceptance of precision fermentation made egg. Front. Sustain. Food Syst..

[89] GFI, Plant-Based Meat (2024).

[90] GFI (2023).

[91] Hosseinkhani N., McCauley J.I., Ralph P.J. (2022). Key challenges for the commercial expansion of ingredients from algae into human food products. Algal Res.

[92] Sogari G., Menozzi D., Hartmann C., Mora C., Sogari G., Mora C., Menozzi D. (2019). Edible Insects in the Food Sector: Methods, Current Applications and Perspectives. [Internet].

[93] GFI, Plant-Based Meat (2023).

[94] GFI, Cultivated meat and seafood (2022).

[95] van Tulleken C. (2023).

[96] Onwezen M.C., Nassar G., Bouma J.A. (2025). Change meat resistance: systematic literature review on consumer resistance to the alternative protein transition. Annu. Rev. Food Sci. Technol..

[97] Abril E.P., Dempsey P.R. (2019). Outcomes of healthy eating Ad campaigns: a systematic review. Prog. Cardiovasc. Dis..

[98] Wadi N.M., Cheikh K., Keung Y.W., Green R. (2024). Investigating intervention components and their effectiveness in promoting environmentally sustainable diets: a systematic review. Lancet Planet. Health.

[99] Nájera Espinosa S., Hadida G., Jelmar Sietsma A., Alae-Carew C., Turner G., Green R. (2024). Mapping the evidence of novel plant-based foods: a systematic review of nutritional, health, and environmental impacts in high-income countries. Nutr. Rev..

