# Supplementary material for: Consumer Acceptability of Animal-Source Food Alternatives: A Literature Review to Support the Transition to Sustainable, Healthy Diets
Source: Curr Dev Nutr. 2026 Aug 11;10(9):109513. doi: 10.1016/j.cdnut.2026.109513 (PMC13545432; doi:10.1016/j.cdnut.2026.109513)
Supplement: Supplementary file 1 [file mmc1.docx]

**Consumer Acceptability of Animal-source Food Alternatives: A Literature Review to Support the Transition to Sustainable, Healthy Diets**

**Supplementary Material**

**Extended Methods**

1. **Full Scopus Search Strings**

The Scopus search was originally designed to facilitate prioritization, as it was expected that resources would be insufficient to screen all results. As such, one search (AC1) focused on only reviews; one focused on non-reviews but required the inclusion of Alt-ASF search terms thought to be the most relevant (AC2); and one focused on non-reviews using a broader set of search terms. In the case of insufficient resources to screen all, we planned to screen all results of AC1 and AC2, and the first 500 of AC3. In practice, all results were screened, and the multi-search approach was effectively moot.

The effective search text was thus:

TITLE-ABS-KEY (acceptability OR acceptance OR desirability OR preference OR appropriateness OR suitability) AND TITLE-ABS-KEY (“alternative protein*” OR “alt protein*” OR “protein alternative*” OR “animal source food alternative*” OR “animal sourced food alternative*” OR “alternative animal source food*” OR “alternative animal sourced food*” OR “novel alternative*” OR “protein substitute*” OR “novel protein*” OR “emerging protein*” OR “plant based alternative*” OR “plant protein*” OR “plant based protein*” OR “plant derived protein*” OR “plant source food*” OR “plant sourced food*” OR “impossible burger*” OR “impossible foods” OR “beyond burger” OR “beyond meat” OR “fungus protein*” OR “fungal protein*” OR “fungus based protein*” OR “fungi protein*” OR “fungi based protein*” OR “mushroom protein*” OR “mushroom based protein*” OR “mycoprotein*” OR “mycelium” OR “quorn” OR “fusarium venenatum” OR “algae based protein*” OR “algae derived protein*” OR “algal protein*” OR “algal based protein*” OR “algae protein*” OR “spirulina” OR “chlorella” OR “microalgae” OR “macroalgae” OR “microalgal protein*” OR “macroalgal protein*” OR “cell based meat*” OR “cell based protein*” OR “lab grown meat*” OR “lab grown protein*” OR “in vitro meat*” OR “in vitro protein*” OR “cultured protein*” OR “cultured meat*” OR “cultivated meat*” OR “cultivated protein*” OR “precision fermentation” OR “fermentation derived” OR “cell based dairy” OR “lab grown dairy” OR “cultivated dairy” OR “synthetic meat*” OR “synthetic dairy” OR “cell based milk*” OR “lab grown milk*” OR “slaughter free meat*” OR “cellular agriculture” OR “insect based food*” OR “insect based protein*” OR “edible insect*” OR “insect powder*” OR “insect flour*” OR “insect protein*” OR “insect derived food*” OR “insect derived protein*” OR “mealworm*” OR “cricket protein*” OR “cricket powder*” OR “cricket flour*” OR “insect meal” OR “edible spider*” OR “grasshopper protein*” OR “grasshopper powder*” OR “grasshopper flour*”) AND PUBYEAR > 1999

The actual search text used for the three separate searches (AC1, AC2, and AC3) is given below; combined, they equal the ‘effective search text’ given above.

**AC1 - Reviews only:**

DOCTYPE ( re ) AND TITLE-ABS-KEY (acceptability OR acceptance OR desirability OR preference OR appropriateness OR suitability) AND TITLE-ABS-KEY (“alternative protein*” OR “alt protein*” OR “protein alternative*” OR “animal source food alternative*” OR “animal sourced food alternative*” OR “alternative animal source food*” OR “alternative animal sourced food*” OR “novel alternative*” OR “protein substitute*” OR “novel protein*” OR “emerging protein*” OR “plant based alternative*” OR “plant protein*” OR “plant based protein*” OR “plant derived protein*” OR “plant source food*” OR “plant sourced food*” OR “impossible burger*” OR “impossible foods” OR “beyond burger” OR “beyond meat” OR “fungus protein*” OR “fungal protein*” OR “fungus based protein*” OR “fungi protein*” OR “fungi based protein*” OR “mushroom protein*” OR “mushroom based protein*” OR “mycoprotein*” OR “mycelium” OR “quorn” OR “fusarium venenatum” OR “algae based protein*” OR “algae derived protein*” OR “algal protein*” OR “algal based protein*” OR “algae protein*” OR “spirulina” OR “chlorella” OR “microalgae” OR “macroalgae” OR “microalgal protein*” OR “macroalgal protein*” OR “cell based meat*” OR “cell based protein*” OR “lab grown meat*” OR “lab grown protein*” OR “in vitro meat*” OR “in vitro protein*” OR “cultured protein*” OR “cultured meat*” OR “cultivated meat*” OR “cultivated protein*” OR “precision fermentation” OR “fermentation derived” OR “cell based dairy” OR “lab grown dairy” OR “cultivated dairy” OR “synthetic meat*” OR “synthetic dairy” OR “cell based milk*” OR “lab grown milk*” OR “slaughter free meat*” OR “cellular agriculture” OR “insect based food*” OR “insect based protein*” OR “edible insect*” OR “insect powder*” OR “insect flour*” OR “insect protein*” OR “insect derived food*” OR “insect derived protein*” OR “mealworm*” OR “cricket protein*” OR “cricket powder*” OR “cricket flour*” OR “insect meal” OR “edible spider*” OR “grasshopper protein*” OR “grasshopper powder*” OR “grasshopper flour*”) AND PUBYEAR > 1999

**AC2 - Only the general Alt-ASF terms, no reviews**

TITLE-ABS-KEY (acceptability OR acceptance OR desirability OR preference OR appropriateness OR suitability) AND TITLE-ABS-KEY (“alternative protein*” OR “alt protein*” OR “protein alternative*” OR “animal source food alternative*” OR “animal sourced food alternative*” OR “alternative animal source food*” OR “alternative animal sourced food*” OR “novel alternative*” OR “protein substitute*” OR “novel protein*” OR “emerging protein*”) AND PUBYEAR > 1999 AND NOT DOCTYPE ( re )

**AC3 - All document types except reviews, all terms except the general ones used in AC2:**

TITLE-ABS-KEY (acceptability OR acceptance OR desirability OR preference OR appropriateness OR suitability) AND TITLE-ABS-KEY (“plant based alternative*” OR “plant protein*” OR “plant based protein*” OR “plant derived protein*” OR “plant source food*” OR “plant sourced food*” OR “impossible burger*” OR “impossible foods” OR “beyond burger” OR “beyond meat” OR “fungus protein*” OR “fungal protein*” OR “fungus based protein*” OR “fungi protein*” OR “fungi based protein*” OR “mushroom protein*” OR “mushroom based protein*” OR “mycoprotein*” OR “mycelium” OR “quorn” OR “fusarium venenatum” OR “algae based protein*” OR “algae derived protein*” OR “algal protein*” OR “algal based protein*” OR “algae protein*” OR “spirulina” OR “chlorella” OR “microalgae” OR “macroalgae” OR “microalgal protein*” OR “macroalgal protein*” OR “cell based meat*” OR “cell based protein*” OR “lab grown meat*” OR “lab grown protein*” OR “in vitro meat*” OR “in vitro protein*” OR “cultured protein*” OR “cultured meat*” OR “cultivated meat*” OR “cultivated protein*” OR “precision fermentation” OR “fermentation derived” OR “cell based dairy” OR “lab grown dairy” OR “cultivated dairy” OR “synthetic meat*” OR “synthetic dairy” OR “cell based milk*” OR “lab grown milk*” OR “slaughter free meat*” OR “cellular agriculture” OR “insect based food*” OR “insect based protein*” OR “edible insect*” OR “insect powder*” OR “insect flour*” OR “insect protein*” OR “insect derived food*” OR “insect derived protein*” OR “mealworm*” OR “cricket protein*” OR “cricket powder*” OR “cricket flour*” OR “insect meal” OR “edible spider*” OR “grasshopper protein*” OR “grasshopper powder*” OR “grasshopper flour*”) AND NOT TITLE-ABS-KEY (“alternative protein*” OR “alt protein*” OR “protein alternative*” OR “animal source food alternative*” OR “animal sourced food alternative*” OR “alternative animal source food*” OR “alternative animal sourced food*” OR “novel alternative*” OR “protein substitute*” OR “novel protein*” OR “emerging protein*”) AND PUBYEAR > 1999 AND NOT DOCTYPE ( re )

*Google Scholar Search Strings*

As Google Scholar does not allow for as flexible of searching (namely, words can anywhere in article or in title, as opposed to being able to limit to the abstract / keywords, and there is no AND/OR Boolean complexity), search terms were simplified to include the general term ‘alternative protein’, plus ‘acceptability’. The search set-up thus looked as follows:

- with **all** of the words acceptability
- with the **exact phrase** alternative protein
- words anywhere in the article
- Return articles **dated** between 2000-

The search was conducted on 24/2/2024 and yielded 4,370 results.

Google Scholar is known to return very large numbers of results, making it infeasible to screen all, but it also enables sorting for relevance. As such, this was used to sort the results, and the first 500 titles were screened. Google Scholar is not very transparent about how its relevance ranking works, but research has shown it is driven heavily by citation counts (1) and occurrence of a search term in an article’s title (2); author or journal name may also play a role (2). As such, there is some risk of bias due to the use of its algorithm, but in the case of a narrative review, where exhaustive inclusion of documents is not necessary, its advantages in terms of efficiency and increasing the likelihood of capturing the most relevant research outweigh this risk. The risk was also mitigated through using both search engines.

2. **Study selection**

Search results from Scopus were imported into Microsoft Excel, and duplicates were removed. Google Scholar does not facilitate easy export of search results, making it difficult to verify duplicates with the Scopus results at the title-screening stage. As such, only those Google Scholar results that passed the title-screening stage were manually exported into an Excel template, at which point duplicates were identified and removed.

Studies were assessed against the eligibility criteria, and only those meeting the criteria were included in the review. First, the title was reviewed for relevance versus inclusion/exclusion criteria. For all relevant titles, the abstract was then reviewed for relevance versus the inclusion/exclusion criteria.

Due to the large number of papers passing the abstract screening (n=410), a prioritization approach was used to meet the aims of the review while keeping the number of full-text screenings and extractions feasible. All 410 studies’ key characteristics (as discernable based on an abstract review) were coded in Excel, then studies were prioritized for full-text review as follows:

1. Twenty-eight of the 29 structured reviews were included, excluding one with no full text available.
2. For those Alt-ASF that had been covered by a recent and strong structured review (i.e., cell-, fungus-, and insect-based foods)^[1]^, we identified papers published after the most recent search covered in those reviews that: included primary data, had acceptability of Alt-ASF as the main focus, covered a general population, and did not have any clear problems with quality. We then included up to 10 of those papers per Alt-ASF type, aiming to fill gaps in the earlier reviews’ coverage.
   a. For fungus-based foods, only three additional studies met these criteria, and all were included.
   b. For cell-based foods, the above reviews only covered cell-based meat, so we included all five studies on cell-based products other than meat that met the abovementioned criteria (excluding the date restriction).
   c. For insect-based foods, 19 additional studies met these criteria. We prioritized nine of them based on coverage of non-Western countries, as the majority of the included reviews were either restricted to Western countries or included mostly studies from Western countries.
3. For the other Alt-ASF categories, plant- and algae-based, we included up to 10 additional papers per Alt-ASF as follows:
   a. We first included all unstructured reviews that focused on acceptability of that Alt-ASF as their main topic of focus, covered global or multicounty general populations, and had no clear quality weaknesses. This included four additional studies on plant-based foods and one on algae-based foods (which also covered other Alt-ASF categories).
   b. We then identified all non-review papers on these foods that met criteria under (2), above.
      i. For plant-based foods, 35 additional studies met these criteria. We prioritized six of them that focused on minimally processed legumes, nuts, or seeds, as this category was not well-covered in the already reviewed literature.
      ii. For insect-based foods, 11 additional studies met these criteria. We prioritized eight of them by excluding three papers that focused on niche topics related to acceptability (positive attitude, social norms, or food-related lifestyles).

Finally, we also included all three additional studies on composite plant-based (plus meat) Alt-ASFs that met the above-mentioned criteria (included primary data, had acceptability of Alt-ASF as their main focus, covered a general population, and did not have any clear problems with quality). This excluded one study on a composite meat-insect protein, as that analysis focused on the acceptance of the insect ingredient, not the hybrid nature of the product.

This approach was designed to ensure sufficient coverage of each Alt-ASF type to support robust conclusions (at least 10 papers per Alt-ASF category, as possible), to prioritize methods that were stronger and upon which broader generalizations could be made (reviews and general population and multi-country samples), and to minimize duplication between original studies and reviews that contained them.

For 65 of the 66 prioritized papers, a full text review was conducted; for the other paper, no full-text version could be found. For papers passing the full-text review, data extraction proceeded as described in Section 3. Title, abstract, and full-text screening, as well as abstract coding and full-text data extraction, were undertaken by one researcher due to limited resources, the narrative (as opposed to systematic) review approach, and the non-sensitive nature of the study topic. The number of excluded studies, with reason, was recorded at each stage.

3. **Data extraction and quality rating**

A standardized form in Microsoft Excel was used to extract data from the included studies during full-text review. The content of this form is included in Table S1. Data extraction was carried out by one researcher. Due to the breadth of the research topic, the narrative review approach, and the diversity of the types of sources consulted, no quality appraisal was conducted. We used the reference management software Zotero to flag any retractions in the papers that passed full-text screening; retracted papers would have been excluded if identified, but none were. During extraction, we flagged any papers with authors with either a disclosed or apparent (based on their affiliation) conflict of interest (n=7 papers), and considered this in the interpretation of results. Given the expectation of very varied types of data and outcomes, including considerable qualitative data, we did not conduct data validation.

4. **Data synthesis: Collating, summarizing, and reporting the findings**

As noted in the main text, to be able to understand the full breadth of the literature, all 410 studies’ key characteristics (as discernable based on an abstract review) were coded in Excel, then analyzed using Stata SE15. This information was used to answer research question 1.

The data collected using the data extraction template were analyzed using descriptive statistics via Microsoft Excel and Stata SE15 to understand the general body of research conducted on the topic. A narrative synthesis was then performed to summarize the included studies and answer the research questions. Given the differences in evidence quality and in types of information reported between review papers and studies collecting primary data, these were synthesized separately: the narrative draws primarily on the results of the reviews, with insights from non-review papers used to complement them by filling key gaps in the reviews (e.g., limited coverage of cell-based products other than meat; limited coverage of algae- and fungus-based products).

The quantitative data that could be extracted through this review was expected to be very heterogenous, but as feasible we sought to extract comparable data on approximate levels of consumer acceptance across food types. We also coded characteristics of the study: types of data collection/analysis methods; types of Alt-ASFs examined; context (region, country income level); and any potential demographic characteristics of the population studied (e.g., gender, age group). Initial coding of this information was done at extraction; at the end of the extraction stage, it was reviewed and refined to ensure comparability across all extracted data.

The main goal of the narrative synthesis was to describe the state of research on the topic of Alt-ASF acceptability and synthesize the results of that research to show how acceptability varies across Alt-ASFs and when compared to ASFs. The synthesis process began by sorting extracted data by the type of Alt-ASF examined among plant-based, fungus-based, algae-based, cell-based, and insect-based. Within each subgroup of Alt-ASF, we focused on the main results of the studies as related to research question 2 and proceeded to synthesize the insights of the literature on that topic. In that synthesis, we considered the suitability of the study methods for answering the research questions. This synthesis was largely qualitative, due to the divergence in outcome variables and study designs, but we included quantitative summaries where feasible. To the extent possible, we tried to examine distinctions between low-income-country and high-income-country settings.

**References**

1.	Rovira, Codina, Guerrero-Solé, Lopezosa. Ranking by Relevance and Citation Counts, a Comparative Study: Google Scholar, Microsoft Academic, WoS and Scopus. Future Internet. 2019 Sep 19;11(9):202. doi:10.3390/fi11090202

2.	Beel J, Gipp B. Google Scholar’s Ranking Algorithm: An Introductory Overview. In: Proceedings of the 12th International Conference on Scientometrics and Informetrics (ISSI’09). Rio de Janeiro: International Society for Scientometrics and Informetrics; 2009. p. 230–41.

3.	Bryant C, Barnett J. Consumer Acceptance of Cultured Meat: An Updated Review (2018–2020). Applied Sciences. 2020 Jan;10(15):15. doi:10.3390/app10155201

4.	Siddiqui SA, Khan S, Ullah Farooqi MQ, Singh P, Fernando I, Nagdalian A. Consumer behavior towards cultured meat: A review since 2014. Appetite. 2022 Dec 1;179:106314. doi:10.1016/j.appet.2022.106314

5.	Pakseresht A, Ahmadi Kaliji S, Canavari M. Review of factors affecting consumer acceptance of cultured meat. Appetite. 2022 Mar 1;170:105829. doi:10.1016/j.appet.2021.105829

6.	Lewisch L, Riefler P. Cultured meat acceptance for global food security: a systematic literature review and future research directions. Agricultural and Food Economics. 2023 Oct 26;11(1):48. doi:10.1186/s40100-023-00287-2

7.	Tsvakirai CZ, Nalley LL, Tshehla M. What do we know about consumers’ attitudes towards cultured meat? A scoping review. Future Foods. 2024 Jun 1;9:100279. doi:10.1016/j.fufo.2023.100279

8.	De Cianni R, Pippinato L, Mancuso T. A systematic review on drivers influencing consumption of edible mushrooms and innovative mushroom-containing products. Appetite. 2023 Mar 1;182:106454. doi:10.1016/j.appet.2023.106454

9.	Mancini S, Moruzzo R, Riccioli F, Paci G. European consumers’ readiness to adopt insects as food. A review. Food Research International. 2019 Aug 1;122:661–78. doi:10.1016/j.foodres.2019.01.041

10.	Mina G, Peira G, Bonadonna A. The Potential Future of Insects in the European Food System: A Systematic Review Based on the Consumer Point of View. Foods. 2023 Jan;12(3):3. doi:10.3390/foods12030646

11.	Puteri B, Jahnke B, Zander K. Booming the bugs: How can marketing help increase consumer acceptance of insect-based food in Western countries? Appetite. 2023 Aug 1;187:106594. doi:10.1016/j.appet.2023.106594 PubMed PMID: 37178930.

12.	Kröger T, Dupont J, Büsing L, Fiebelkorn F. Acceptance of Insect-Based Food Products in Western Societies: A Systematic Review. Front Nutr. 2022 Feb 21;8. doi:10.3389/fnut.2021.759885

13.	Alhujaili A, Nocella G, Macready A. Insects as Food: Consumers’ Acceptance and Marketing. Foods. 2023 Jan;12(4):4. doi:10.3390/foods12040886

14.	Florença SG, Guiné RPF, Gonçalves FJA, Barroca MJ, Ferreira M, Costa CA, et al. The Motivations for Consumption of Edible Insects: A Systematic Review. Foods. 2022 Nov 15;11(22):3643. doi:10.3390/foods11223643

**Table S1. Contents of the data extraction form**

| **Questions** | **Response options** |
| --- | --- |
| First author name (last, first) | Open text |
| All other authors | Open text |
| Are any authors affiliated with industry or have declared COIs? | Choose: yes, no |
| If yes, specify the affiliation(s) / COIs | Open text |
| Year of publication | Number |
| Title | Open text |
| DOI | alphanumeric code |
| Type of document | Choose: peer-reviewed journal article, book chapter, technical report, other: specify |
| Journal name | Open text |
| Study aims and/or research questions | (copy paste from Introduction/abstract) |
| Country/ies of study | Open text |
| Country income group | Using World Bank classification: HIC, UMIC, LMIC, LIC |
| Other aspects of geographical context | If relevant. Urban / rural, etc. |
| Alt-ASF focus | Open text |
| Alt-ASF focus (categorical) | Choose: Plant-based; Fungus-based; Algae-based; Cell-based; Insect-based |
| Alt-ASF definitions: How do they define or describe alt-ASFs in general or the specific Alt-ASF examined? | (text; either copy from paper or summarize in 1-2 sentences) |
| What aspect of affordability does the study cover? (general) | Open text |
| Are there any other key aspects of the paper/study topic's framing? | E.g. a particular motivation, intended use, or tone |
| Any other comments on introduction, framing, focus, and research questions. | Open text |
| Method summary : | Choose: 1) qualitative; 2) quantitative; 3) mixed-methods; 4) modelling; 5) review; 6) synthesis/thought piece; 7) other: specify |
| Additional details on methodology | Open text. |
| Does the study include data collected from people? | Choose: yes/ no. |
| Who was assessed/ interviewed (sample population description) ? | Open text. |
| Specify the data collection tool used (select all that apply): | Choose: survey, modelling, observation, interviews, focus group, document analysis, other: specify |
| Sample population description | Open text. |
| Sample size | How many interviews, surveys, etc (description text, 1-2 sentences) |
| Sampling - how did they recruit respondents/participants? | Open text. |
| Sampling - code | 1) Snowball, 2) Convenience, 3) existing sample pool (e.g. online study), 4) Random (including stratified random) sampling, 5) census (all); 6) purposive; 77) other specify; 9) none specified |
| Percent of the sample that is female | Percentage |
| Age breakdown of the sample | Text ; |
| For secondary data, specify the source and type | Open text. |
| Comment on any weaknesses seen in the methods | Open text. |
| Any other comments on methods | Open text |
| Is price or affordability the main focus of the paper? | Choose: yes/ no. |
| What is the time focus of the analysis? | Choose: Past; present; future; other |
| What specific affordability outcomes does the article talk about? | Choose all that apply: sales price; production cost; cost of diet; comparison to income/purchasing power; qualitative description; other: specify |
| Key findings on affordability (summary) | Summarize in 1-2 sentences |
| Key findings on affordability (detail) | Text from the paper |
| Any other key findings of relevance | Open text. |
| Risks/challenges for affordability identified | Open text. |
| Opportunities/benefits for affordability identified | Open text. |
| Are there any insights related to gender? If so, summarize | Open text. |
| Are there any insights related to other subgroup characteristics? If so, summarize | Open text. |
| Are there any particularly relevant figures/tables? | Choose: Yes, No |
| If applicable, any other comments on the results | Open text. |
| What is the overall tone of the discussion/paper vis-à-vis the alt-ASF in question? | Choose: positive; negative; neutral/none; mixed (pros and cons) |
| Any authors' comments on the future prospects for Alt-ASFs | Open text. |
| Any authors' comments on barriers to adoption of Alt-ASFs / negative aspects of Alt-ASFs | Open text. |
| Any authors' comments on facilitators to adoption of Alt-ASFs / positive aspects of Alt-ASFs | Open text. |
| Are there future research or gap identified, usually mentioned in the discussion section of the paper? | Choose: Yes, No |
| If yes to previous question, what are they (copy/paste text) | Open text |
| Are there any recommendations identified, usually mentioned in the discussion section of the paper? | Choose: yes, no |
| If yes to previous question, what are they (copy/paste text) | Open text |
| Does the paper also include results on livelihoods? | Choose: Yes, No |
| Does the paper also include results on acceptability? | Choose: Yes, No |

1. This applied to **cell-based meat** (3–7), with the most recent search being mid-2023; to **fungus-based foods** (8), searched in early 2022; and **insect-based foods** (9–14), with the most recent search being in 2022.
